# Supplementary material for: Sport and dance interventions for healthy young people (15–24 years) to promote subjective well-being: a systematic review
Source: BMJ Open. 2018 Jul 15;8(7):e020959. doi: 10.1136/bmjopen-2017-020959 (PMC6082460; doi:10.1136/bmjopen-2017-020959)
Supplement: Supplementary file 1 [file bmjopen-2017-020959supp001.pdf]

OVID MEDLINE Search Strategy

1. MeSH descriptor: [well being]

2. well-being

3. wellbeing

4. “young people”.mp or youth.mp or adolescent*.mp

5. sport/ or sport.mp.

6. “physical activity”.mp or “physical activity”/

7. Exercise*.mp.

8. “physical exertion”.mp.

9. dance*.mp.

10. game*.mp.

11. team.mp.

12. bike.mp.

13. cycl*.mp.

14. cheerlead*.mp.

15. equestrian.mp.

16. swim*.mp.

17. gym* .mp.

18. sail*.mp.

19. canoe*.mp.

20. kayak*

21. bloodsport*.mp.

22. boxing.mp

23. “martial arts”.mp.

24. fitness.mp.

25. ballet.mp.

26. choreograph*

27. “work-out”.mp.

28. (1 or 2 or 3) and (4) and (or 5 or 6 or 7 or 8 or 9 or 10 or 11 or 12 or 13 or 14 or 15 or 16 or 17 or 18, or 19 or 20 or 21 or 22 or 23 or 24 or 25 or 26 or 27)

29. tournament.mp

30. match.mp

31. competition.mp

32. festival.mp

33. battle.mp

34. league.mp

35. team*.mp

36. theatre*.mp

37. event*.mp

38. meet*.mp

39. field*.mp

40. fan.mp

41. play*.mp

42. athlet*.mp

43. attend*.mp

44. spectat*.mp

45. particpat*.mp

46. perform*.mp

47. 28 and (29 or 30 or 31 or 32 or 33 or 34 or 35 or 36 or 37 or 38 or 39 or 40 or 41 or 42 or 43 or 44 or 45 or 46)

48. Quality of life.mp. or “Quality of Life”/ Life

49. Anxiety/ or anxiety.mp.

50. self-esteem.mp.

51. loneliness/ or lonel. mp.

52. life adj satisfaction.mp.

53. happiness.mp.

54. worthwhileness.mp.

55. 47 and (48 or 49 or 50 or 51 or 52 or 53 or)

56. limit 54 to humans and all young people or adolescents
