# Supplementary material for: Sport and dance interventions for healthy young people (15–24 years) to promote subjective well-being: a systematic review
Source: BMJ Open. 2018 Jul 15;8(7):e020959. doi: 10.1136/bmjopen-2017-020959 (PMC6082460; doi:10.1136/bmjopen-2017-020959)
Supplement: Supplementary file 2 [file bmjopen-2017-020959supp002.pdf]

Table of excluded studies

Authors	Year	Reason for exclusion
Adie JW, Duda JL, Ntoumanis N.	2008	Intervention
Ahola R, Pyky R, Jämsä T, Mäntysaari M, Koskimäki H, Ikäheimo TM, Huotari ML, Röning J, Heikkinen HI, Korpelainen R.	2013	Study design
Altintas A, Asci FH, Kin-Isler A, Guven-Karahan B, Kelecek S, Ozkan A, Yilmaz A, Kara FM.	2014	Population
Anamaria Constantinescu.	2013	Outcome
Aphamis G, Giannaki CD, Tsouloupas CN, Ioannou Y, Hadjicharalambous M.	2015	Outcome
Aramendi Jauregui P, Bujan Vidales K, Arburua Goyeneche R.	2014	Intervention
Arts Council England	2006	Year
Bamford, C.	2015	Study design
Barton J, Pretty J.	2010	Study Design
Beresford B, Clarke S.	2009	Year
Berntsson LT, Ringsberg KC.	2014	Intervention
BHF National Centre	2014	Study design
Black Country Consortium Ltd	2014	Outcome
Blazy L, Amstel S	NR	Study design
Booker CL, Skew AJ, Kelly YJ, Sacker A.	2015	Intervention
Booker CL, Skew AJ, Sacker A, Kelly YJ.	2014	Intervention
Boyer EM.	2007	Year
Brand S, Gerber M, Beck J, Hatzinger M, Pühse U, Holsboer-Trachsler E.	2010	Intervention
Brassai L, Piko BF, Steger MF.	2011	Intervention
Broďáni J, Spišiak M, Paška Ľ.	2015	Intervention
Brown DR, Carroll DD, Workman LM, Carlson SA, Brown DW.	2014	Population
Buckinghamshire County Council	NR	Study design
Burgess G, Grogan S, Burwitz L.	2006	Population

Casey MM, Harvey JT, Telford A, Eime RM, Mooney A, Payne WR.	2014	Population
Castillo I, Duda JL, Alvarez MS, Merce J, Balaguer I.	2011	Population
Chatzisarantis NLD, Hagger MS.	2007	Intervention
Chen LH, Kee YH.	2008	Intervention
Chen LH, Kee YH, Chen MY.	2015	Outcome
Crossick G, Kaszynska P.	2016	Intervention
Dance is Public Health	2015	Study design
Daniels E, Leaper C.	2006	Intervention
D'anna C, Rio L, Paloma FG.	2015	Intervention
De Bruin AP, Woertman L, Bakker FC, Oudejans RRD.	2009	Intervention
Department of Health, Physical Activity, Health Improvement and Protection	2011	Year
Department of Culture, Arts and Leisure	2009	Year
Di Luzio SS, Procentese F, Guillet-Descas E.	2014	Not available from the British Library
Doerksen SE, Elavsky S, Rebar AL, Conroy DE.	2014	Intervention
Englefield L, Cunningham D, Mahoney A, Stone T, Torrance H.	2016	Outcome
Eime RM, Harvey JT, Brown WJ, Payne WR.	2010	Study design
Falconer C.	2010	Year
Findlay LC, Bowker A.	2009	Population
Fløtnes IS, Nilsen TIL, Augestad LB.	2011	Intervention
Fujiwara D, Kudrna L, Cornwall T, Laffan K, Dolan P.	2015	Outcome
Fujiwara D, Kudrna L, Dolan P.	2014	Population
Fujiwara D, Kudrna L, Dolan P.	2014	Outcome
Fujiwara D, MacKerron G.	2015	Intervention
Gardner SM, Komesaroff P, Fensham R.	2008	Intervention
Geyer J.	2013	Intervention

Gondoh Y, Sensui H, Kinomura S, Fukuda H, Fujimoto T, Masud M, Nagamatsu T, Tamaki H, Takekura H.	2009	Population
Hagensen KP.	2015	Population
Hidalgo-Rasmussen CA, Ramírez-López G, Martín AH-S.	2013	Intervention
HM Government	2016	Outcome
Holland J.	2012	Year
Ivanović M, Milosavljević S, Ivanović U.	2015	Outcome
Jago R, Sebire SJ, Davies B, Wood L, Banfield K, Edwards MJ, Powell JE, Montgomery AA, Thompson JL, Fox KR.	2015	Population
Jalaludin B, Maxwell M, Saddik B, Lobb E, Byun R, Gutierrez R, Paszek J.	2012	Population
Jančiauskas R.	2012	Population
Jelalian E, Hart CN, Mehlenbeck RS, Lloyd-Richardson EE, Kaplan JD, Flynn-O'Brien KT, Wing RR.	2008	Outcome
Jonsdottir IH, Börjesson M, Ahlborg Jr. G.	2011	Population
Jowett GE.	2014	Outcome
Kaczmarek LD, Drążkowski D.	2014	Intervention
Kallings LV, Leijon M, Hellénius M-L, Ståhle A.	2008	Population
Kantor RM, Grimes GR, Limbers CA.	2015	Population
Karadağ Çaman Ö, Özcebe H.	2011	Intervention
Kardefelt-Winther D.	2014	Intervention
Kavetsos G, Szymanski S.	2010	Study design
Kelly P, Matthews A, Foster C.	2012	Year
Kelly NR, Mazzeo SE, Evans RK, Stern M, Thacker LF, Thornton LM, Laver JH.	2011	Population
Kern ML, Waters LE, Adler A, White MA.	2015	Intervention
Khan Y, Taghdisi MH, Nourijelyani K.	2015	Intervention
Kim J, Suh W, Kim S, Gopalan H.	2012	Intervention
Kipp LE, Weiss MR.	2013	Population
Knab AM, Nieman DC, Sha W, Broman-Fulks JJ, Canu WH.	2012	Population

Knifsend CA.	2015	Population
Komlosi, E	2014	Intervention
Kort-Butler LA, Hagewen KJ.	2011	Intervention
Kowert R, Vogelgesang J, Festl R, Quandt T.	2015	Intervention
Lafrenière MA, Vallerand RJ, Donahue EG, Lavigne GL.	2009	Intervention
Laure P, Binsincer C.	2009	Population
Laure P, Binsincer C.	2009	Population
Laurendeau J.	2014	Intervention
Lazaridou A, Kalogianni C.	2013	Outcome
Le Menestrel S, Perkins DF.	2007	Intervention
Lee AJY, Lin WH.	2007	Outcome
Lee BW, Leeson PRC.	2015	Intervention
Leggett, Diane K.	2010	Intervention
Lestan KA, Eržen I, Golobič M.	2014	Population
Leversen I, Danielsen AG, Birkeland MS, Samdal O.	2012	Study Design
Lieber SB, Redberg RF, Blumenthal RS, Gandhi A, Robb KJ, Mora S.	2012	Population
Liu M, Wu L, Ming Q.	2015	Study Design – Systematic Review
Lopez-Walle J, Balaguer I, Castillo I, Tristan J.	2012	Population
Lorger M, Mrakovic S, Hraski M.	2012	Population
LSE Housing and Communities team	2015	Study design
Lu FJH, Hsu YW.	2013	Intervention
Lupu E, Petrescu A.L.	2012	Study Design
Mack DE, Wilson PM, Gunnell KE, Gilchrist JD, Kowalski KC, Crocker PR.	2012	Study design
Madison G, Paulin J, Aasa U.	2013	Population
Maffulli N, Longo UG, Spiezia F, Denaro V.	2010	Intervention
Magnusson M, Hallmyr Lewis M, Smaga-Blom M, Lissner L, Pickering C.	2014	Study Design

Mäkinen M., Lindberg N., Komulainen E., Puukko-Viertomies L.-R., Aalberg V., Marttunen M.	2015	Population
Mancini, JA; Bowen, GL; O'Neal, CW; Arnold, AL	2015	Intervention
Mansfield L, Kay T, Anokye N, Fox-Rushby J.	2015	Study design
Martin-Albo, J; Nunez, JL; Dominguez, E; Leon, J; Tomas, JM	2012	Population
Maugendre M., Spitz E.	2011	Study Design
McDade-Montez, Elizabeth; Wallander, Jan; Elliott, Marc;Grunbaum,Jo Anne; Tortolero, Susan; Cuccaro, Paula; Schuster, Mark A.	2015	Intervention
McGee, R., Williams, S., Howden-Chapman, P., Martin, J. and Kawachi, I	2006	Study design
McMahon E.M., Corcoran P., O'Regan G., Keeley H., Cannon M., Carli V., Wasserman C., Hadlaczky G., Sarchiapone M., Apter A., Balazs J., Balint M., Bobes J., Brunner R., Cozman D., Haring C., Iosue M., Kaess M., Kahn J.-P., Nemes B., Podlogar T., Poštuvan V., Sáiz P., Sisask M., Tubiana A., Värnik P., Hoven C.W., Wasserman D.	2016	Population
Medeiros M.D., De Castro Filho J.A.	2014	Not available from the British Library
Mental Health Foundation	2013	Study design
Merrill R.M., Aldana S.G., Bowden D.E.	2010	Population
Mihaela, Cristuţă Alina	2012	Population
Mochon, D; Norton, MI; Ariely, D	2008	Population
Mohan S., Smith C.A., Corriveau N.L., Kline-Rogers E., Jackson E.A., Eagle K.A., Goldberg C., Durussel-Weston J.	2012	Intervention
Moljord I., Moksnes U.K., Eriksen L., Espnes G.A.	2011	Study Design
Molina J.J.M., Castillo A.S., De La Serrana H.L.G., Díaz M.Z.	2009	Population
Molina-Garcia J, Castillo I, Queralt A	2011	Study Design
Moutão J., Alves S.M., Monteiro D., Cid L.	2015	Population
Nicholls L., Lewis A.J., Petersen S., Swinburn B., Moodie M., Millar L.	2014	Intervention
Noack P., Kauper T., Benbow A.E.F., Eckstein K.	2013	Study Design
Oliver, S.	2009	Year
Optimity Advsors	2016	Population

Orkibi H., Ronen T., Assoulin N.	2014	Population
Papaioannou A.G., Appleton P.R., Torregrosa M., Jowett G.E., Bosselut G., Gonzalez L., Haug E., Ertesvaag V., Zourbanos N.	2013	Population
Peng W., Crouse J.	2013	Outcome
Pérez Ugidos, Guillermo; Laíño, Fernando, A.; Zelarayán, Julio; Márquez, Sara	2014	Intervention
Phillips G Renton A Moore DG Bottomley C Schmidt E Lais S Yu G Wall M Tobi P Frostick C Clow A Lock K Petticrew M Hayes R	2012	Population
Physical Activity Council	2016	Intervention
Piqueras J.A., Kuhne W., Vera-Villarroel P., Van Straten A., Cuijpers P.	2011	Study Design
Play Wales	2012	Year
Precor	NR	Study design
Proctor C., Tsukayama E., Wood A.M., Maltby J., Eades J.F., Linley P.A.	2011	Intervention
Public Health England	2015	Study design
Pyky, R; Jauho, AM; Ahola, R; Ikaheimo, TM; Koivumaa-Honkanen, H; Manysaari, M; Jamsa, T; Korpelainen, R	2015	Intervention
Reding, Frank N; Grieve, Frederick; Derryberry, W. Pitt; Paquin, Anthony R	2011	Outcome
Riley A., Anderson-Butcher D.	2012	Population
Rössler R., Donath L., Verhagen E., Junge A., Schweizer T., Faude O.	2014	Study Design – Systematic Review
Rotheram-Borus M.J., Swendeman D., Becker K.D.	2014	Population
Ryan., K, Mind	2015	Intervention
Sagar, S.S.	2007	Year
Sagatun, A., Søgaard, A.J., Bjertness, E., Selmer, R. and Heyerdahl, S	2007	Study design
Sage, L; Kavussanu, M	2010	Population
Salehi, A; Harris, N; Sebar, B; Coyne, E	2015	Population
Schlarb A.A., Schwedler V., Feichtinger P.	2012	Study Design
Schmiedeberg C., Schröder J.	2016	Population

Schuch F.B., Pinto S.S., Bagatini N.C., Zaffari P., Alberton C.L., Cadore E.L., Silva R.F., Kruel L.F.M.	2014	Population
Schulz, KH; Meyer, A; Langguth, N	2012	Population
Schwanen, T; Wang, DG	2014	Population
Sekot A.	2013	Population
Sellakumar G.K.	2015	Intervention
Shaffer-Hudkins, Emily	2012	Population
Shiue, I	2016	Population
Sidoti E., Paolini G., Tringali G.	2010	Population
Sigvartsen J., Gabrielsen L.E., Abildsnes E., Stea T.H., Omfjord C.S., Rohde G.	2016	Study Design
Sjögren K., Hansson E.E., Stjernberg L.	2011	Population
Skianis, V.	2013	Intervention
Slough Borough Council	NR	Year
Smyth, W.	NR	Population
Snyder A.R., Martinez J.C., Bay R.C., Parsons J.T., Sauers E.L., McLeod T.C.V.	2010	Study Design
Spandler H Mckeown M Roy A Hurley M	2013	Population
Spengler, Sarah; Woll, Alexander	2013	Population
Sport and Recreation Alliance	2012	Year
Sport and Recreation Alliance	2016	Study Design
Stein C., Fisher L., Berkey C., Colditz G.	2007	Population
Stenseng, Frode; Forest, Jacques; Curran, Thomas	2015	Population
StreetGames	2016	Study design
Stubbe J.H., de Moor M.H.M., Boomsma D.I., de Geus E.J.C.	2007	Population
Student Sport Ireland	2016	Outcome
Suendermann, S.,	2015	Population
Sztankovics A.	2013	Population
Tanimaru J.H., Dos Santos A.L.P.	2016	Study Design

Taylor, P., Davies, L., Wells, P., Gilbertson, J. & Tayleur, W.	2015	Study design
Tharenos C.L., Santorino D.	2009	Not available from the British Library
The Department of Culture, Arts and Leisure	2009	Year
Thøgersen-Ntoumani C., Ntoumanis N.	2006	Population
Thomley B.S., Ray S.H., Cha S.S., Bauer B.A.	2011	Population
TOP Foundation	2014	Study design
UK Community Foundations	2012	Year
Urmston, E.	2012	Year
Urmston, E.	2013	Population
Vilela C., Gomes A.R.	2015	Intervention
Wall M., Hayes R., Moore D., Petticrew M., Clow A., Schmidt E., Draper A., Lock K., Lynch R., Renton A.	2009	Study design
Watson, B., Lashua, B., Trevorrow, P.	2016	Outcome
Whitehead, S.H.	2005	Year
Wicker, P; Coates, D; Breuer, C	2015	Population
Wicker, P; Frick, B	2015	Population
Williams K., Davis III O., Gittelman M., Pomerantz W.J.	2006	Population
Williams, G. & Jacques, K.	NR	Population
Woodall, J; White, J; South, J	2013	Population
Yamada K., Kawata Y., Nakajima N., Hirosawa M.	2012	Outcome
Zook K.R., Saksvig B.I., Wu T.T., Young D.R.	2014	Outcome
Zullig, Keith J.; White, Rebecca J.	2011	Population
