# Supplementary material for: Sport and dance interventions for healthy young people (15–24 years) to promote subjective well-being: a systematic review
Source: BMJ Open. 2018 Jul 15;8(7):e020959. doi: 10.1136/bmjopen-2017-020959 (PMC6082460; doi:10.1136/bmjopen-2017-020959)
Supplement: Supplementary file 3 [file bmjopen-2017-020959supp003.pdf]

Published Literature Data Extraction Form

Reviewer Initials:

**Title, Author, year**	
**Study objectives**	
**Study design**	
**Method of allocation to study group**	
**Outcomes and measures used (relevant to review)** (Include scale(s) used and time-points)	
**Intervention** (brief description of the intervention used)	
**Details of analysis** (Include type of analysis i.e. quantitative/qualitative/mixed, and method and/or process of analysis e.g. thematic analysis/statistical analysis, any subgroup analysis and any methods used in the treatment of missing data)	
**Participants included (at baseline and follow up in each group)** (Source/recruitment, eligible and selected, number, age restrictions, exclusions, gender)	
**Intervention(s) and comparison group(s)** (Type, content, intervener, duration, method, mode or timing of delivery)	
**Results** (Key numerical results including proportions experiencing relevant outcomes in each group, means, medians, standard deviations, ranges and effect sizes with precision estimates e.g. confidence intervals/ p values whether or not significant [if P values are not reported this should be stated]. For qualitative data what categories/themes were found, results drawn by authors and evidence provided. Identify any inadequately reported missing data	
**Protected characteristics** (Methods and findings that relate to protected characteristics [age, sex, gender reassignment, sexual orientation, disability, race, religion, pregnancy/maternity, marriage/civil partnerships] and income and/or socio-economic status.	
**Limitations identified**	

**Review conclusions**<br>(for each comparison made)	
**Conflicts of interest and sources of funding**	
**Ethical procedures reported**	
**Grade/CERQual Rating**	

GRADE and CERQual for judging certainty / quality of evidence

Quantitative: Grade

**Type of evidence**	Randomized trial = high<br>Observational study = low<br>Any other evidence = very low
**Decrease grade if**<br>**(Each quality criteria can reduce the quality by one or, if very serious, by two levels.)**	• Serious or very serious limitation to study quality (e.g. Important inconsistency; major uncertainty about directness; imprecise or sparse data; high probability of reporting bias
**Increase grade if**	• Strong evidence of association—significant relative risk of > 2 (< 0.5) based on consistent evidence from two or more observational studies, with no plausible confounders (+1)<br>• Very strong evidence of association—significant relative risk of > 5 (< 0.2) based on direct evidence with no major threats to validity (+2)<br>• Evidence of a dose response gradient (+1)<br>• All plausible confounders would have reduced the effect (+1)
**Grade Rating / Range**	High quality evidence<br>Moderate quality evidence<br>Low quality evidence<br>Very low quality evidence

Qualitative: CERQual

**Increase confidence if**	• Study is well designed with few limitations<br>• Evidence applicable to context (perspective or population, phenomenon of interest, setting) specified in objectives<br>• Findings/conclusions supported by evidence and provide convincing explanation for patterns found<br>• Data supporting findings is rich and good quality
**Decrease confidence if**<br>(Each quality criteria can reduce the quality by one or, if very serious, by two levels)	• Serious or very serious limitations in design or conduct of the study<br>• Evidence is not relevant to the study objectives<br>• Findings/conclusions are not supported by the evidence<br>• Data is poor quality and inadequate to support findings
**CERqual Confidence Rating / Range**	**High confidence** It is highly likely that the review finding is a reasonable representation of the phenomenon of interest<br>**Moderate confidence** It is likely that the review finding is a reasonable representation of the phenomenon of interest<br>**Low confidence** It is possible that the review finding is a reasonable representation of the phenomenon of interest<br>**Very low confidence** It is not clear whether the review finding is a reasonable representation of the phenomenon of interest.

Grey literature data extraction tool

Part 1. Project details

Author details
Record of the authors' details, date of publication, title of the report, publisher and place of publication.
Project aims
Include aims and objectives for the project.
Project partners
Record the organisations involved in project delivery. Who is the lead delivery partner who managed the intervention?
Commissioner(s) and funding sources
Who funded the project?
Type of arts or sport intervention
E.g. music, singing, dance etc.
Project description
For how long did the intervention run? How many sessions, episodes or events were delivered? What were the delivery dates? Record the key components, activities and events. Include details of equipment needed to run the intervention and staff competencies of those delivering it. Where did the project take place? Include geographical location and type of setting, e.g. museum, college, sports centre. It is important to record any special conditions, such as incentives or access to prestigious venues that may have affected participants' experiences of the project.
Target population
Who was the target population? Include age, gender, ethnicity, demographic details, health conditions and localities if relevant. How were participants recruited to the intervention? E.g. referral process or is it self-selecting? How many people actually took part?
Project costs
Record details of project costs, including costs per participant, and costs to participants, if reported.

Part 2: Evaluation details

Evaluation aims and objectives
What was the rationale for the evaluation? What key outcomes and impacts were prioritised for evaluation. What questions did the evaluation seek to address? Did it build on previous work, e.g. a theory of change/logic model/evidence review/research study or previous evaluation?
Conducting the evaluation
Who conducted the evaluation? Who managed it and was responsible for any changes in the design or responding to adverse events?
Type of evaluation and evaluation design
E.g. basic monitoring, process evaluation, quantitative, qualitative, mixed methods etc.
Evaluation budget
What resources were set aside for evaluation? What was the duration of evaluation funding, if this was received?
Data collection procedures
Provide details of quantitative and qualitative data collection procedures.
Sampling, selection and recruitment of participants
How were participants selected for data collection, including qualitative interviews, focus groups and case studies? How many actually people took part?
Evaluation timeline
When were the data collected?
Ethics and consent
What were the ethical considerations for the evaluation? Was the anonymity of participants be protected? Were the participants particularly vulnerable? Was formal ethics approval obtained?
Data analysis
How were the data analysed? Were there any biases in data analysis and reporting?
Key findings
What wellbeing outcomes were reported? How was wellbeing reflected in qualitative themes?
Findings from process evaluation
What broader impacts or learning were recorded?

Reference

Adapted from Public Health England Arts and Health Evaluation Framework
https://www.gov.uk/government/uploads/system/uploads/attachment_data/file/496230/PHE_Arts_and_Health_Evaluation_FINAL.pdf
