# Supplementary material for: Sport and dance interventions for healthy young people (15–24 years) to promote subjective well-being: a systematic review
Source: BMJ Open. 2018 Jul 15;8(7):e020959. doi: 10.1136/bmjopen-2017-020959 (PMC6082460; doi:10.1136/bmjopen-2017-020959)
Supplement: Supplementary file 4 [file bmjopen-2017-020959supp004.pdf]

# Quality checklist: Quantitative evidence of intervention effectiveness

**How to use this checklist:** This checklist is to be used to indicate if a specific study has been well designed, appropriately carried out and analysed, i.e. the confidence we can have in the results of whether an intervention was effective. This should be used for the summary table, to make brief comments on the risk of bias of each study. In turn, the overview of the study limitations will help to inform the quality of the overall body of evidence.

| Evidence quality of intervention effectiveness / study limitations | | | | |
|---|---|---|---|---|
| **1. Was the evaluation well-designed?** | **Yes** | **No** | **Can't tell** | **N/A** |
| • **Fidelity:** The extent to which the intervention was delivered with fidelity is clear - i.e. if there is a specific intervention which is being evaluated, this has been well reproduced.<br>• **Measurement**: The measures are appropriate for the intervention's anticipated outcomes and population.<br>• Participants completed the same set of measures once shortly before participating in the intervention and once again immediately afterwards<br>• An 'intent-to-treat' design was used, meaning that all participants recruited to the intervention participated in the pre/post measurement, regardless of whether or how much of the intervention they received, even if they dropped out of the intervention (this does not include dropping out of the study- which may then be regarded as missing data)<br>• **Counterfactual**:<br>• Assignment to the treatment and comparison group was at the appropriate level (e.g., individual, family, school, community)<br>• The comparison condition provides an appropriate counterfactual to the treatment group. Consider:<br>  ○ Participants were randomly assigned to the treatment and control group through the use of methods appropriate for the circumstances and target population OR sufficiently rigorous quasi-experimental methods (regression discontinuity, propensity score matching) were used to generate an appropriately comparable sample through non-random methods<br>  ○ The treatment and comparison conditions are thoroughly described. | | | | |

| **2. Was the study carried out appropriately? including appropriate sample** | **Yes** | **No** | **Can't tell** | **N/A** |
|---|---|---|---|---|
| • **Representative:** The sample is representative of the intervention's target population in terms of age, demographics and level of need. The sample characteristics are clearly stated.<br>• There is baseline equivalence between the treatment and comparison group participants on key demographic variables of interest to the study and baseline measures of outcomes (when feasible)<br>• **Sample size**: The sample is sufficiently large to test for the desired impact. This depends most importantly on the effect size, however a suggestion could be e.g. a minimum of 20 participants have completed the measures at both time points within each study group.<br>• **Attrition:** A minimum of 35% of the participants completed pre/ post measures. Overall study attrition is not higher than 65%.<br>• The study had clear processes for determining and reporting drop-out and dose. Differences between study drop-outs and completers were reported if attrition was greater than 10%.<br>• The study assessed and reported on overall and differential attrition<br>• **Equivalence:** Risks for contamination of the comparison group and other confounding factors have been taken into account and controlled for in the analysis if possible:<br>  ○ Participants were blind to their assignment to the treatment and comparison group<br>• There was consistent and equivalent measurement of the treatment and control groups at all points when measurement took place.<br>• **Measures**: The measures used were valid and reliable. This means that the measure was standardised and validated independently of the study and the methods for standardization were published. Administrative data and observational measures may also have been used to measure programme impact, but sufficient information was given to determine their validity for doing this.<br>• Measurement was independent of any measures used as part of the treatment.<br>• In addition to any self-reported data (collected through the use of validated instruments), the study also included assessment information independent of the study participants (eg, an independent observer, administrative data, etc). | | | | |

| 3. Was analysis appropriate? | Yes | No | Can't tell | N/A |
|---|---|---|---|---|
| • The methods used to analyse results are appropriate given the data being analysed (categorical, ordinal, ratio/ parametric or non-parametric, etc) and the purpose of the analysis.<br>• Appropriate methods have been used and reported for the treatment of missing data. | | | | |
| **4. Is the evidence consistent?** | | | | |
| • Are the findings made explicit?<br>• Is there adequate discussion of the evidence both for and against the researcher's arguments?<br>• Has the researcher discussed the credibility of their findings (e.g. triangulation, respondent validation, more than one analyst)?<br>• Are the findings discussed in relation to the original research question? | | | | |
