# Supplementary material for: Sport and dance interventions for healthy young people (15–24 years) to promote subjective well-being: a systematic review
Source: BMJ Open. 2018 Jul 15;8(7):e020959. doi: 10.1136/bmjopen-2017-020959 (PMC6082460; doi:10.1136/bmjopen-2017-020959)
Supplement: Supplementary file 5 [file bmjopen-2017-020959supp005.pdf]

Table of Subjective Wellbeing Measures used in Included Studies

Measurement tool	Outcome measuring	Description	Scoring/ interpretation	Validity & Reliability
Rosenberg's Self-Esteem Scale	Self-esteem	10-item scale that measures global self-worth by measuring both positive and negative feelings about the self. The scale is believed to be uni-dimensional. All items are answered using a 4-point Likert scale format ranging from strongly agree to strongly disagree. Five of the items have positively worded statements and five have negatively worded ones. The scale measures state self-esteem by asking the respondents to reflect on their current feelings.	Range: 0-30<br>15- 25 normal range;<br>below 15 low self-esteem.	The original sample for which the scale was developed in the 1960s consisted of 5,024 high school juniors and seniors from 10 randomly selected schools in New York State and was scored as a Guttman scale. The scale generally has high reliability: test-retest correlations are typically in the range of .82 to .88, and Cronbach's alpha for various samples are in the range of .77 to .88.
Athlete Burnout Questionnaire (Raedeke & Smith 2001)	Athletes level of Burn out	15 item assessing 3 dimensions of burnout:<br>-Emotional/physical exhaustion<br>-Reduced sense of accomplishment<br>-Sport devaluation<br><br>The stem for each item is “How often do you feel this way?” Each response is scored on a 5-point Likert scale: “almost never” (1), “rarely” (2), “sometimes” (3), “frequently” (4), “almost always” (5).	Combined scores from each item for a dingle global indicator<br>(higher the score the higher the level of burnout)	Raedeke and Smith (2001) and Cresswell and Eklund (2006) demonstrated reliability and validity both in and out of North America
Beck Depression Inventory first published in 1961, revised in 1978 (BDI-1A) and then 1996 (BDI-II)	Depression (presence and degree. NOT a diagnostic instrument)	21-question multiple-choice self-report inventory for adolescents and adults. Evaluates 21 symptoms of depression (15 on emotions, 4 on behavioural changes, 6 on somatic symptoms).<br>The 21 items cover sadness, pessimism, past failure, self-dislike, self-criticism, suicidal thoughts or wishes, crying,	0–9 not depressed<br>10–18 mild-moderate depression<br>19–29 moderate-severe<br>30–63 severe<br><br>According to paper:<br>0–9 normal<br>10–15 low	Beck reviewed 11 studies and the BDI was capable of discriminating between groups that contrasted in level of depression. Beck’s original paper reported an internal consistency studies demonstrated a correlation coefficient of .86 for the test items, and the Spearman-

		agitation, loss of interest, indecisiveness, worthlessness, loss of energy, changes in sleeping patterns, irritability, changes in appetite, difficulty concentrating, tiredness or fatigue, and loss of interest in sex.<br>Time to Administer: 5-10 minutes	16–23 medium<br>24+ depressive	Brown correlation for the reliability of the BDI yielded a coefficient of .93.<br>Criticisms; BDI-IA only addresses six out of the nine DSM-III criteria for depression, self-reported (reporting bias), questionnaire therefore the way administered could affect outcome e.g. social desirability. If pt has a physical illness the physical symptoms such as fatigue may score higher but not reflect depression.
Subjective Exercise Experiences Scale (SEES)	Measuring 3 dimensions; positive well-being, psychological distress, and fatigue	"By circling a number on the scale below each of the following items, please indicate the degree to which you are experiencing each feeling now, at this point in time, after exercising". Each item rated on a 7-point Likert scale: 1 (Not at all) - 7 (Very much so).<br>12 item scale (4 items per dimension): great, awful, drained, positive, crummy, exhausted, strong, discouraged, fatigued, terrific, miserable, and tired.	The Items (4 items per dimension) are summed to create a summary score for Positive Well-Being, Psychological Distress and Fatigue. Therefore each dimension has a possible score up to 28, the higher the number the higher the association with the trait.	Validity and reliability have been reported for other groups (McAuley & Courneya, 1994; Rudolph & Kim, 1996).
Positive Affect Negative Affect Schedule (PANAS) (Watson, Clark, & Tellegen, 1988)	Hedonic Well-Being/ the intensity associated with both positive and negative dimensions of global affect	20-item self-report instrument. Rate each using a 5-point Likert scale ranging from 1 (Not At All or Very Slightly) to 5 (Very Much).<br><br>PANAS for Children (PANAS-C): 30-item measure (15 positive affect and 15 negative affect items). Indicate how often they have felt interested, sad, and so on during the "past few weeks" on a 5-point Likert scale ranging from 1 (very slightly or not at all) to 5 (extremely).	Positive Affect Score:<br>range from 10 – 50, with higher scores representing higher levels of positive affect.<br>Negative Affect Score:<br>ranges from 10 – 50, with lower scores representing lower levels of negative affect.<br><br>PANAS-C:<br>Summation scores for positive affect and negative affect range from 10 to75 each.	Reliability and Validity reported by Watson (1988) was moderately good. For the Positive Affect Scale, the Cronbach alpha coefficient was 0.86 to 0.90; for the Negative Affect Scale, 0.84 to 0.87. Over a 8-week time period, the test-retest correlations were 0.47-0.68 for the PA and 0.39-0.71 for the NA. The PANAS has strong reported validity with such measures as general

				distress and dysfunction, depression, and state anxiety.<br><br>PANAS-C has demonstrated good convergent and discriminant validity in adolescent samples
Self-Esteem Scale (SES)	Self-esteem	10 items, and the total score ranges from 10 to 40.	Higher scores = higher self-esteem	NR
Profile of Mood States (POMS) scale	Mood & mindfulness	7 subscales (tension, anger, fatigue, depression, vigor, confusion, and mood related to self-esteem) with 40 adjectives that describe mood.<br><br>Original: 65 adjectives rated on 5-point scale<br>0= not at all; 1=a little; 2=moderately; 3=quite a bit; 4=extremely<br><br>Short Form (POMS-SF): 30-item consisting of 30 adjectives rated on a 5-point scale ranging from 0 (not at all) to 4(extremely).	Higher scores (POMS Total Mood Disturbance (TMD)) = more negative current mood states<br><br>POMS-SF:<br>Responses are summed (with positive items reverse scored) to provide a TMD score (range 0–100), as well as subscale scores for 6 mood states (each ranging 0–20): Tension-Anxiety, Depression-Dejection, Anger-Hostility, Vigor-Activity, Fatigue-Inertia, and Confusion-Bewilderment.	High internal consistency of subscales and validity for original POMS scale
WHOQOL-BREF Scale	QoL	The World Health Organization Quality of Life (WHOQOL). WHOQOL-BREF is a shorter version containing 26 items (1 from each of the 24 facets in the WHOQOL-100 plus 2 items from the Overall quality of Life and General Health facet) measuring these domains: physical health, psychological health, social relationships, and environment.	QoL profile with 4 domain scores plus overall perception of QoL and overall perception of health. Higher scores = higher QoL.	developed by the WHOQOL Group with<br>fifteen international field centres, simultaneously, in an attempt to develop a quality of life assessment that<br>would be applicable cross-culturally
Schulte Grid	Attention	A Schulte table (8*8 grid) is a square that consists of 64 squares of the same size (1 × 1 cm), with one of 64 random numbers from 1 to 64. When tested, individuals are required to figure out the numbers in the	Less time represents higher level of attention	NR

		order from 1 to 64, and read out the numbers loud at the same time. Timing starts with 1 and ends with 64.		
Perceived stress scale (PSS) (Cohen et al, 1983).<br><br>Chinese Perceived Stress Scale (CPSS)	Stress	10-items measuring the degree to which events are appraised as stressful during the past month. Items rated on a Likert scale from 0 (never) to 4 (very often).<br>Items designed to tap how unpredictable, uncontrollable, and over-loaded respondents find their lives. The scale also includes several direct queries about current levels of experienced stress.<br><br>CPSS-14 questions	Responses summed to give a total score ranging from 0 to 40 (CPSS: 0-56).<br><br>Higher composite scores indicate greater perceived stress.	The PSS is the most widely used psychological instrument for measuring the perception of stress.<br>CPSS-10 showed a stable two-factor structure with satisfactory internal consistency and construct validity (Siu-man Ng, 2013)
Inventory of Positive Psychological Attitudes	Positive worldview, Confidence in Life and Self (two sub-scales: Life Purpose and Satisfaction (LPS) and Self-Confidence During Stress (SCDS)).	32-item, 7-point Likert self-report scale. Example questions: Life Purpose and Satisfaction Section: My daily activities are -<br>Response: not a source of satisfaction to a source of satisfaction (7 pt scale)<br>Self-Confidence During Stress Section: When there is a great deal of pressure being placed on me - Response: I get tense to I remain calm (7 pt scale).	Each score is calculated as a mean; possible scores ranging from 1 to 7. VERY LOW: 1.00 TO 2.49<br>MEDIUM LOW: 2.50 TO 3.99<br>MEDIUM HIGH: 4.00 TO 5.49<br>VERY HIGH: 5.50 TO 7.00	It has been shown to possess adequate reliability and construct validity in samples of undergraduate college students.
Resilience Scale	self-regulatory skills (degree of individual resilience)	25-item covering 5 factors of resilience; meaningful life (purpose); perseverance; self-reliance; equanimity; and coming home to yourself (existential aloneness). Items scored on a 7-point scale from 1 (disagree) to 7 (agree)	Possible scores ranging from 25 to 175. Higher scores reflect greater resilience.	The scale has internal consistency, reliability, and concurrent validity and has been recommended as the best instrument for measuring resilience in adolescents (Ahern et al, 2006).
Child Acceptance Mindfulness Measure	self-regulatory skills (mindfulness)	25-item measure assessing the degree to which children and adolescents observe internal experiences, act with awareness,	A total score is calculated by reverse scoring negatively worded items and summing the item total. Range in scores from 0 to 100.	The CAMM has demonstrated good internal consistency and concurrent validity with negative correlations to measures of

		and accept internal experiences without judging them.	Higher scores indicate higher levels of acceptance and mindfulness.	cognitive suppression and psychological inflexibility in a study of 606 middle school students (Coyne, Cheron & Ehrenreich, 2008)
State Trait Anger Expression Inventory-2TM (Spielberger, 1999)	self-regulatory skills (experience, expression, and control of anger)	Designed for people aged 16 years and older.<br>57-item self-report tool with a 4-point Likert response format. The instrument is categorized into subscales that reflect state anger (3 subscales), trait anger (2 subscales), and anger expression ().<br><br>Study reported in used sub scales measuring anger expression. Anger expression was conceptualized as having 3 major components: anger-out (outward expression of anger), anger-in (anger suppression), and anger control (attempts to control expression of anger)	For each scale, summation scores range from 8 to 32.<br><br>Higher the score = stronger association. Higher Anger-in = more negative anger expression, higher anger-out = more negative anger expression, higher anger control = better anger control.	Strong reliability and validity. STAXI-2 has been shown to be a suitable instrument to measure both the experience and the expression of anger in both general and clinical populations (Lievaart, Franken, Hovens, 2014).
Friendship Quality Questionnaire	Peer support	assess the quality of children's and early adolescents' relationships with their best friends according to five dimensions: companionship, conflict, help/aid, security and closeness.	NR	A confirmatory factor analysis, used to evaluate the factor structure of this instrument, demonstrated that these scales represented distinct, but related, domains of friendship.<br>Assessments of reliability indicated the high level of internal consistency within each dimension. The validity of the scale was indicated by the observation of higher ratings for (a) mutual friends than for non-mutual friends, and (b) for stable friends than for non-stable friends.

Exercise Confidence Survey	Self-efficacy	8 items each on a 10 pt likert scale (I know I can, to 10 I know I cannot)	Total the numbers circled and the higher the score, the less likely you are to stick with your exercise program.	
-5 item Subscale of the Intrinsic Motivation Inventory	Need Satisfaction (sport competence)	5 items – rated on 7 pt Likert scale (1 strongly disagree – 7 strongly agree)	Higher score = agree more	Each has shown adequate psychometric properties with adolescent athletes in similar studies testing SDT (Amarose & Anderson-Butcher 2007)
-6 item Scale (developed by Hollembeak & Amorose, 2005)	Need Satisfaction (need for autonomy)	the measure asks respondents to indicate the amount of choice or control they have when participating in their current sport.<br>6 items - rated on a range from 1 (not at all true) to 5 (completely true)		
-Sport oriented version of Richer & Vallerand's Feelings of Relatedeness Scale	Need Satisfaction (need for relatedeness)	rate the extent they agree with a series of 10 adjectives describing their relationships with members of their sport team. Range from 1 (do not agree at all) to 7 (very strongly agree)		
-Anger (16 item questionnaire)	Anger	questionnaires were developed by DIPAS (Defense Institute of Physiology and Allied Sciences), New Delhi, India. Every item amongst all the questionnaires measures the tested domain on the weighted scores of responses from 0 (never) to 3 (almost always).	Questionnaires were scored by adding the weighted (0 to 3) scores of each item.<br><br>Sense of well-being: The lesser the score the better is the sense of well-being	These questionnaires were chosen as they are valid for Indian population, reliable and specific to measure the tested psychological domains.
-Trait anxiety (40 item questionnaire)	Anxiety			
-Depression (10 item questionnaire)	Depression (incl. depressed mood, guilt, difficulty in sleeping, decision making, work and interests)			
-Subjective well-being (50 item questionnaire)	Subjective WB (incl. the ability to			

	develop persons' potential; work productivity and creativity; build strong and positive relationships with others)			
The Swedish version of a 10-item General Self-Efficacy Scale (GSES)	Self-efficacy	The Swedish version of the 10-item General Self-Efficacy Scale (GSES), developed by Koskinen-Hagman, Schwartzer and Jerusalem. Original version used a 4-point Likert scale, but a pilot test demonstrated that was too limited to detect variations in participants' responses. This scale was extended to a 6-point Likert scale	The total score is calculated by finding the sum of all items. The total score ranges between 10and 60, with a higher score indicating more self-efficacy. This paper argues that lower score indicates a higher perceived GSE. (note original scale says higher score = higher S-E)	GSES is correlated to emotion, optimism, work satisfaction. Negative coefficients for depression, stress, health complaints, burnout, and anxiety. Internal reliability for GSE = Cronbach's alphas between .76 and .90
Swedish version - Social Barriers to Exercise Self-Efficacy Questionnaire (SPBESQ)	Behaviour specific self-efficacy	6-point Likert scale. Response ranges from 1 'not true' to 6 'absolutely true'.10 items (3 for support barriers and 7 for social barriers). Examines content of intervention and specific behavioural changes.	A lower score appears to suggest a higher perceived SSBES in this paper (note lack of clarity re: direction of effect)	NR
