# Supplementary material for: Sport and dance interventions for healthy young people (15–24 years) to promote subjective well-being: a systematic review
Source: BMJ Open. 2018 Jul 15;8(7):e020959. doi: 10.1136/bmjopen-2017-020959 (PMC6082460; doi:10.1136/bmjopen-2017-020959)
Supplement: Author's manuscript [file bmjopen-2017-020959.draft_revisions.pdf]

BMJ Open

BMJ Open is committed to open peer review. As part of this commitment we make the peer review history of every article we publish publicly available.

When an article is published we post the peer reviewers' comments and the authors' responses online. We also post the versions of the paper that were used during peer review. These are the versions that the peer review comments apply to.

The versions of the paper that follow are the versions that were submitted during the peer review process. They are not the versions of record or the final published versions. They should not be cited or distributed as the published version of this manuscript.

BMJ Open is an open access journal and the full, final, typeset and author-corrected version of record of the manuscript is available on our site with no access controls, subscription charges or pay-per-view fees (http://bmjopen.bmj.com).

If you have any questions on BMJ Open's open peer review process please email
info.bmjopen@bmj.com

BMJ Open

Sport and dance interventions for healthy young people (15-24 years) to promote subjective wellbeing: A systematic review

Journal:	*BMJ Open*
Manuscript ID	bmjopen-2017-020959
Article Type:	Research
Date Submitted by the Author:	06-Dec-2017
Complete List of Authors:	Mansfield, Louise; Brunel University, Sport and Education<br>Kay, Tess; Brunel University London, Life Sciences<br>Meads, Catherine; Anglia Ruskin University, Faculty of Health, Social Care and Education<br>Grigsby-Duffy, Lily; Brunel University<br>Lane, Jack; University of Brighton Faculty of Education and Sport<br>John, Alistair; Brunel University<br>Daykin, Norma; University of Winchester<br>Dolan, Paul; London School of Economics and Political Science<br>Testoni, Stefano; London School of Economics and Political Science<br>Julier, Guy; University of Brighton Faculty of Education and Sport<br>Payne, Annette; Brunel University<br>Tomlinson, Alan; University of Brighton Faculty of Education and Sport<br>Victor, Christina; Brunel University, Social Work & Community/Public Health
Keywords:	Sport, Dance, Wellbeing

SCHOLARONE™
Manuscripts

Sport and dance interventions for healthy young people (15-24 years) to promote subjective wellbeing: A systematic review

Corresponding Author: Dr Louise Mansfield, Department of Life Sciences, Division of Sport Health and Exercise Sciences, Brunel University London, Kingston Lane, Uxbridge, UB8 3PH, UK

Email: louise.mansfield@brunel.ac.uk

Tel: 01895 267561

Co-authors: Professor Tess Kay[1], Professor Catherine Meads[2], Lily Grigsby Duffy[1,] Jack Lane[4], Dr Alistair John[1], Professor Norma Daykin[3], Professor Paul Dolan[5], Stefano Testoni[5], Professor Guy Julier[4], Dr Annette Payne[1], Professor Alan Tomlinson[4], Professor Christina Victor[6]

[1.] Life Sciences, Brunel University London, Uxbridge, UK

[2.] Nursing and Midwifery, Anglia Ruskin University, Cambridge, UK

[3.] Health and Wellbeing, University of Winchester, Winchester, UK

[4.] Arts and Culture, University of Brighton, Brighton, UK

[5.] Social Policy, The London School of Economics and Political Science, London, UK

[6.] Clinical Sciences, Brunel University London, Uxbridge, UK

Word count, excluding title page, abstract, references, figures and tables = 4, 370

For peer review only - http://bmjopen.bmj.com/site/about/guidelines.xhtml

ABSTRACT

Objective: to review and assess the effectiveness of sport and dance participation on subjective wellbeing outcomes among 15-24 year olds.

Design: Systematic review

Methods: We searched for studies published in any language between January 2006 and September 2016 on PsychINFO, Ovid MEDLINE, Eric, Web of Science (Arts and Humanities Citation Index, Social Science and Science Citation Index), Scopus, PILOTS, CINAHL, SPORTDiscus, and International Index to Performing Arts. Additionally, we searched for unpublished (grey) literature via an online call for evidence, expert contribution, searches of key organisation websites and the British Library EThOS database, and a keyword Google search. Published studies of sport or dance interventions for healthy 15-24-year olds where subjective wellbeing was measured were included. Studies were excluded if participants were paid professionals or elite athletes, or if the intervention was clinical sport/dance therapy. Two researchers extracted data and assessed strength and quality of evidence using criteria in the What Works Centre for Wellbeing methods guide and GRADE, and using standardised reporting forms. Due to clinical heterogeneity between studies, meta-analysis was not appropriate. Grey literature which was a final evaluation report on empirical data relating to sport or dance interventions was included.

Results: Eleven out of 6587 articles were included (7 RCTs and 1 cohort study, and three unpublished grey evaluation reports). Published literature suggests meditative physical activity (yoga and Baduanjin Qigong) and group-based or peer supported sport and dance has some potential to improve subjective wellbeing. Grey literature suggests sport and dance improve subjective wellbeing but identify negative feelings of competency and capability. The quality of published evidence on sport and dance interventions to enhance subjective wellbeing is low.

Conclusions: Meditative activities, group and peer supported sport and dance may promote subjective wellbeing enhancement in youth. Evidence is limited. Better designed studies are needed.

Registration: PROSPERO (CRD42016048745).

For peer review only - http://bmjopen.bmj.com/site/about/guidelines.xhtml

STRENGTHS AND LIMITATIONS OF THIS STUDY

- A comprehensive search strategy was used including searches of published and unpublished data, and study selection was carried out by two reviewers independently.
- Data extraction and quality assessments were conducted using standardised forms, independently by two reviewers.
- The focus on a specific target age group may have excluded evidence from studies that have aggregated data across younger and older age groups in their analysis.
- Meta-analysis was not possible due to the heterogeneity of study interventions and outcomes

For peer review only

INTRODUCTION

Governments and international organisations acknowledge subjective wellbeing (SWB) as a policy goal.[1-3] The international focus on measuring SWB is gaining recognition in some aspects of UK sport,[4-5] dance,[6] and physical activity policy.[7] SWB describes wellbeing in terms of the good and bad feelings arising from what people do and how they think. [8] Good feelings include happiness, joy, contentment, and excitement while sadness, worry, stress, and anxiety are examples of more negative feelings. People's experiences also involve a sense of purpose (e.g. worthwhileness, meaningfulness) and pointlessness (e.g., futility, boredom). Since 2011, SWB measured as satisfaction with life, worthwhileness, happiness, and anxiety has been included in UK population surveys conducted by the Office of National Statistics (ONS).[9] Links between sports and cultural activities and SWB have been reported and sport engagement is included in national-level data collection and analysis.[10] Significant associations have been found between engagement in sport, the arts and enhanced SWB as measured by life satisfaction.[11] Yet, it is acknowledged that SWB is a complex concept, with no single agreed definition or measure.[12] The term SWB is used synonymously with a wide range of concepts including self-esteem, self-efficacy, self-determination, resilience, quality of life, mood enhancement, positive mental health, life satisfaction, worthwhileness and happiness.[13] Measures of SWB use various scales that demonstrate wellbeing as multidimensional (e.g. The Warwick and Edinburgh Mental Wellbeing Scale,[14] Rosenberg's Self-Esteem Scale,[15] The Profile of Mood States,[16] Better understanding of the effects of sport and

For peer review only - http://bmjopen.bmj.com/site/about/guidelines.xhtml

dance on a range of SWB measures is therefore central to informing policy development, programme delivery and measurement and evaluation of sport and dance interventions to enhance wellbeing.

The ESRC funded What Works Centre for Wellbeing[17] initiative has commissioned evidence reviews in key areas including Culture, Sport and Wellbeing. Following consultation with stakeholders,[18] four topics were identified for systematic review between 2015 and 2018. This paper reports the findings of the second systematic review topic; sport and dance interventions for healthy young people (15-24 years) to promote subjective wellbeing.

Sport refers to forms of physical activity either casually or formally organised in which people take part for fitness, health and wellbeing, social relationships or competition.[19] Sport includes a wide range of individual and group activities including jogging, running, cycling, martial arts, yoga, team games and athletics. Dance refers to the rhythmic movements and sequences of steps usually set to music. Both sport and dance organisations identify young people as a key target group for engagement in physical activity to enhance wellbeing. The evidence, however, is theoretically and methodologically diverse and less attention has been given to children and adolescents. Existing evidence reviews on sport have tended to focus on physical rather than mental health or wellbeing outcomes[20-22] or they have examined the effect of exercise in populations with specific mental health conditions such as depression[23] and anxiety.[24-25] Dance-related reviews of evidence have examined the effectiveness of dance therapy on psychological and physical health and wellbeing outcomes in cancer patients,[26] for schizophrenia[27] and on depression.[28] A review of reviews on physical activity and mental health in children and adolescents identified an association between physical activity and positive wellbeing outcomes connected to reduced depression and anxiety, and enhanced self-esteem and cognitive function.[29] No systematic review to date has focused on sport and dance interventions in healthy young people (15-24 years) to promote subjective wellbeing.

For peer review only - http://bmjopen.bmj.com/site/about/guidelines.xhtml

METHODS

The protocol for this systematic review was registered with the International Prospective Register of Systematic Reviews (PROSPERO) on 3rd October 2016 (Registration number CRD42016048745). The review follows the Preferred Reporting Items for Systematic Reviews and Meta-Analysis (PRISMA) guidelines. [30]

Inclusion criteria

Inclusion criteria were any comparative studies investigating any form of sport or dance compared to no sport or dance, usual routine, or comparing pre- and post-test scores in healthy young people aged between 15-24 years and measuring any form of subjective wellbeing (see table 1). We included studies worldwide from countries economically similar to the UK (using OECD – DAC list of country development; http://www.oecd.org/dac/stats/daclist.htm) or with study populations similar in terms of socioeconomic status. Studies could be fully published (with search dates of 2006-2016) or grey literature (with search dates of 2013-2016). Shorter timescales for grey literature search ensured a focus on finding recent relevant studies that had not yet been published. Grey literature was included if it was a final evaluation or report on empirical data, had the evaluation of sport-related or dance interventions as the central objective, and included details of authors (individuals, groups, or organisations).

Exclusion criteria

Published studies were excluded if participants were paid professionals or elite athletes, or if the intervention was sport or dance therapy delivered in a clinical setting to for rehabilitation purposes. We did not include studies of walking as there is existing review level evidence on the health and wellbeing benefits of this activity. Grey literature was excluded if it did not meet the criteria for inclusion on date, format of reporting, type of data and details of authorship. Eligibility criteria are summarised in table 1.

For peer review only

For peer review only - http://bmjopen.bmj.com/site/about/guidelines.xhtml

Data sources and search strategy

We searched for empirical studies published between January 2006 and September 2016 on the following databases: PsychINFO, Ovid MEDLINE, Eric, Web of Science (Arts and Humanities Citation Index, Social Science and Science Citation Index), Scopus, PILOTS, CINAHL, SPORTDiscus, and International Index to Performing Arts. There were no language restrictions.

Electronic databases were searched using a combination of Medical Subject Headings (MeSH) and free text terms. An example of the OVID MEDLINE search strategy used can be found in appendix 1. All database searches were based on this strategy but were appropriately revised to suit each database.

Additionally, reference lists of all relevant reviews[31-36] from the last five years were hand-searched to identify additional relevant empirical evidence. We also carried out a search for up-to-date UK unpublished (grey) literature completed between 2013 and 2016 via: (i) an online call for evidence on the What Works Wellbeing website between October and November 2016; (ii) contacting known experts in the field for recommendations of sport or dance sector reviews or repositories to search; (iii) a review of key sector websites; (iv) a search of the British Library EThOS website for unpublished PhD dissertations and; (v) reviewing the titles of the first 100 results in a Google search with the use of key terms ('sport' AND 'physical activity' AND 'dance' AND 'wellbeing' AND 'young people'). 'Physical Activity' was included as a search term because it is used by the sector when reporting on sport and dance activities.

Study selection

Two reviewers independently screened the titles and abstracts of all studies identified by the search strategy for their eligibility. Where it was not clear from the title and abstract whether a study was relevant, the selection criteria were independently applied to the full article to confirm its eligibility. Where two independent reviewers did not agree in their primary judgements they discussed the conflict and attempted to reach a consensus. If they could not agree then a third member of the

For peer review only - http://bmjopen.bmj.com/site/about/guidelines.xhtml

review team considered the full paper and a majority decision was made. Appendix 2 lists excluded studies and reasons for exclusions.

For peer review only

Table 1 Eligibility criteria for selecting studies

PICOS criteria	Inclusion	Exclusion
Participants	▪ Participants were to be 15-24 years of age.<br>▪ Studies from countries economically similar to the UK (i.e. other high-income countries with similar economic systems) or with study populations that have similar socioeconomic status to UK.	▪ Participants with a health condition diagnosed by a health professional.<br>▪ Participants who were paid professionals or elite athletes.<br>▪ Participants in clinically-based sport and dance interventions.
Intervention	▪ Participatory sport and dance interventions including watching and performing.<br>▪ Including sport-related and dance therapy offered to enhance wellbeing in healthy young people.	▪ Clinical sport-based or dance therapy.<br>▪ Sport and dance for clinical procedures such as surgery, medical tests, and diagnostics<br>▪ Walking
Comparison	▪ No sport or dance, usual routine i.e. inactive comparator, or historical/time-based comparator i.e. pre-post study design.	
Outcomes	▪ Subjective wellbeing using any recognised method or measure	
Study design	▪ Empirical research: either quantitative, qualitative, or mixed methods, outcomes, or process evaluations<br>▪ Grey literature: if it was a final evaluation or report on empirical data, had the evaluation of sport-related or dance interventions as the central objective, and included details of authors (individuals, groups, or organisations)<br>▪ Published studies published between 2006-2016.<br>▪ Grey literature and practice surveys published between 2013-2016	▪ Discussion articles, commentaries or opinion pieces not presenting empirical or theoretical research<br>▪ Grey literature if it did not have details of authorship

For peer review only - http://bmjopen.bmj.com/site/about/guidelines.xhtml

Data extraction

Two review authors independently extracted data using a standardised form (Appendix 3). Discrepancies were resolved by discussion and consensus. Where agreement could not be reached a third review author considered the paper and a majority decision was reached. The following data were extracted: (1) evaluation design and objectives (the interventions studied and control conditions used, including detail where available on the intervention content, dose and adherence, ethics); (2) sample (size, representativeness, reporting on drop-out, attrition and details of participants including demographics and protected characteristics where reported); (3) the outcome measures (the scales used and the collection time-points, independence, validity, reliability, appropriateness to wellbeing impact questions); (4) analysis (assessment of methodological quality/limitations); (5) results and conclusions; (6) the presence of possible conflicts of interest for authors. In order to capture project details in the grey literature we used an adapted version of the Public Health England Arts and Health Evaluation Framework[37] and extracted: project aims; costs; commissioners, partners and funding sources; intervention details; population and; reported outcomes. Where available, evaluation details (aims, objectives, budget, procedures, timeline, data analysis, and findings) were also extracted.

Our protocol included for us to contact the authors of articles if the required information could not be extracted and was essential for the interpretation of their results but we did not need to do this.

Quality assessment

To assess the methodological quality of the included published studies, two review authors independently applied the quality checklist for quantitative studies based on the Early Intervention Foundation checklist and detailed in the What Works Centre for Wellbeing methods guide[38] (Appendix 4). The checklist was used to indicate if a specific study had been well designed, appropriately carried out and properly analysed. A summary of quality scores is presented in table 2.

For peer review only - http://bmjopen.bmj.com/site/about/guidelines.xhtml

Table 2 Quality checklist scores of included published studies: What Works Centre for Wellbeing checklist

**Authors (date)**	**Evaluation Design**							**Sample**															**Analysis**		TOTAL SCORE: STUDY
	Participants completed the same set of measures before and after intervention	Appropriate random assignment to treatment and control conditions	Group assignment was at the appropriate level (e.g. individual, community)	An intent-to-treat design was used	The treatment and comparison conditions are thoroughly described	The extent to which the intervention was delivered with fidelity is clear	Appropriate comparison condition	The sample is representative of the target population and characteristics stated	The sample is sufficiently large to test for the desired impact (min 20 per group)	There is a clear process for determining and reporting drop-out and dose	Overall study attrition no higher than 65%	Baseline equivalence between treatment and comparison groups	Confounding factors controlled for	Participants blinded to group assignment	Consistent and equivalent measurement	Clear processes for determining and reporting drop-out and dose	Assessed and reported on overall and differential attrition	Appropriate measures were used	Measures used were valid and reliable	Measurement independent of treatment measures	Measurement was blind to group	Included assessment information independent of the participants e.g. independent observer	Appropriate methods used to analyse	Appropriate methods used for the treatment of missing data	
Akandere & Demir (2011)[39]	x	x	x	x	x	x			x		x	x	x		x		x	x	x	x			x	x	17
Amorose et al (2009)[46]	x					x			x						x	x		x	x	x			x		9
Kanojia et al (2013)[40]	x	x	x		x	x	x		x			x			x			x	x	x			x		13
Kim & Kim (2007)[41]	x	x	x				x	x	x		x	x			x			x	x	x			x		13
Li et al (2015)[42]	x	x	x	x	x	x	x	x	x	x	x	x			x	x	x	x	x	x	x		x	x	21
Lindgren et al (2011)[43]	x	x	x			x		x	x	x	x	x	x		x	x	x	x	x	x		x	x	x	19
Noggle et al. (2012)[44]	x	x	x	x	x		x	x			x	x			x		x	x	x	x			x		15
Staiano et al (2013)[45]	x	x	x		x	x	x	x		x	x	x				x	x	x		x			x	x	16

For peer review only - http://bmjopen.bmj.com/site/about/guidelines.xhtml

We then employed the Grading of Recommendations Assessment, Development and Evaluation working group methodology (GRADE) schema for judging strength and quality of evidence on wellbeing overall from sport and dance interventions. Four categories of evidence are used in GRADE; high, moderate, low, or very low. Applying GRADE, RCT studies were initially judged as high quality and sound observational studies as low quality. Evidence was downgraded for methodological limitations, inconsistent findings, sparse data, indirect evidence and reporting bias. Evidence was graded upwards if there was a large magnitude of effect or a dose-response gradient. The PHE Arts for Health and Wellbeing Evaluation Framework[37] was used to judge the quality of the grey literature in terms of the appropriateness of the evaluation design, the rigour of the data collection and analysis and precision of reporting.

Data synthesis

Due to heterogeneity of interventions and wellbeing outcomes between studies, a meta-analysis was not appropriate. We reported the findings narratively. Summaries of the characteristics of the included studies were organised in a tabular form (see table 3) and present information on the participants (number and characteristics), intervention and comparison conditions, outcomes and measure used, study design, conclusions, and study limitations. Summaries of the results (number of participants, mean scores and standard deviations [SD] for each outcome measure at each measurement point, and a summary of the results. No studies reported confidence intervals and so these have not been included) are presented in table 4 and synthesised in the text according to sport/dance intervention type and wellbeing outcomes.

For peer review only

For peer review only - http://bmjopen.bmj.com/site/about/guidelines.xhtml

Table 3 Characteristics of included studies

**Published literature**						
**Authors (Date)** ***Country***	**Numbers of Participants**	**Participant Description**	**Intervention/comparison**	**Outcomes and measures used** ***Measurement times***	**Study Design**	**Limitations (risk of bias)**
Akandere and Demir (2011)[39] *Turkey*	N = 120	Gender: 50% female<br>Age: 20 - 24<br>Ethnicity: NR	12-week dance training intervention<br><br>Comparison: no intervention	-Depression (Beck Depression Scale)<br><br>*Before and after 12 week dance intervention*	RCT	• Only one measure used<br>• Small population<br>• Sample already had dance knowledge<br>• Participant details not clearly reported<br>• Baseline levels of depression differ in groups
Amorose et al (2009)[46] *USA*	N = 93	Members of a competitive club volleyball programme in Midwestern U.S.<br>Gender: Female<br>Age: 13-18 (M=15.78 yrs).<br>Ethnicity: Mostly Caucasian (90.6%).	Followed a cohort of female adolescent volleyball players through a season of competitive volleyball games (approx. 4 months)<br><br>Comparison: Time (before vs. after)	1.Need Satisfaction<br>- Sport competence (5 item Subscale of the Intrinsic Motivation Inventory)<br>- Need for autonomy (6 item Scale: Hollembeak & Amorose 2005)<br>- Need for relatedeness (10 item Richer & Vallerand's Feelings of Relatedeness Scale)<br>2.Well being<br>-Self-esteem (10 item Rosenberg's Self-Esteem Scale)<br>- Burnout (15 item Athlete Burnout Questionnaire)<br><br>*1-2 wks before competitive season starts and post season (1-2 wks before the last official game / ~4m after start of season)*	Cohort	• Sample bias: one club in Western U.S., one sport. All females. Mostly Caucasian<br>• Selection bias: only those that agreed to volunteer. Drop out not reported<br>• Study design: no control group. Only 2 time points looked at<br>• Did not assess social contextual factors e.g. coaching behaviour
Kanojia et	N = 50	Gender: Female	Yoga practiced for 35-40	-Anger (16 item questionnaire)	RCT	• Drop out not reported

For peer review only - http://bmjopen.bmj.com/site/about/guidelines.xhtml

al (2013)[40]<br>*India*		Age: 18-20<br>Ethnicity: NR (study conducted in the Department of Physiology, Lady Hardinge Medical College and Smt. Sucheta Kriplani Hospital, New Delhi, India)	minutes/day x 6 days/week for the duration of 3 menstrual cycles. Training given by qualified instructor<br><br>Comparison: no intervention	-Trait anxiety (40 item questionnaire)<br>-Depression (10 item questionnaire)<br>-Subjective wellbeing (50 item questionnaire)<br>Questionnaires were developed by DIPAS (Defense Institute of Physiology and Allied Sciences)<br><br>*At the beginning and after completion of three menstrual cycles*		• Recruitment methods not reported<br>• Not possible to double blind<br>• *Consistent findings*
Kim and Kim (2007)[41]<br>*Korea*	N = 277	Gender:<br>Age: 17 - 22 (M=20.6)<br>Ethnicity: Korean high school (n = 45) and undergraduate students (n = 232) volunteers.	1 of 4 40 minute exercise sessions: aerobic exercise, body conditioning, hip-hop dancing, and ice skating	-Mood (Subjective Exercise Experiences Scale: measuring 3 dimensions; positive wellbeing, psy distress, and fatigue)<br><br>*Before and after the exercise session.*	RCT	• Data based on one session only.
Li et al (2015)[42]<br>*China*	N = 222	College students<br>Gender: 82.5% female<br>Age: 18-25 (Mage 20.78)<br>Ethnicity: NR. (Recruited from college in China)	Baduanjin exercise 1hr/day 5x week x12 weeks<br><br>Comparison: usual exercise	- Self-esteem (Self-esteem Scale [SES])<br>- Mood / mindfulness (Profile of Mood States [POMS] scale)<br>- QoL (WHOQOL-BREF)<br>-<br>- Stress (Chinese Perceived Stress Scale [CPSS])<br>-Self-symptom intensity (SCL-90 scale)<br><br>*Baseline (before start), at the end of the intervention (week 13), 12-week follow up*	RCT	• Not blinded<br>• Participants recruited from one medical university<br>• Greater proportion of female participants<br>• Small effect size<br>• *Excellent protocol adherence*<br>• *No significant loss to follow up*

For peer review only - http://bmjopen.bmj.com/site/about/guidelines.xhtml

				(week 25)		
Lindgren et al (2011)[43]<br>*Sweden*	N = 110	Physically inactive students from secondary schools in low SES areas.<br>Gender: female<br>Age: ~15<br>Ethnicity: NR	Empowerment-based exercise intervention programme. Sessions included exercise (45 minutes at a moderate level) and discussion (15 minutes). During the discussion time, topics such as healthy lifestyles were addressed.<br>2x wk for 6 months<br><br>Comparison: waiting list	- Self-efficacy (Swedish version of a 10-item General Self-efficacy Scale)<br>- Behaviour changes (Social Barriers to Exercise Self-efficacy Questionnaire)<br><br>*Once at the start of the programme and once at end (6 months)*	RCT	• Small sample size<br>• High dropout rate
Noggle et al (2012)[44]<br>*USA*	N= 51	Students at a public high school in rural western Massachusetts.<br>Age: Average age 17	A Kripalu-based yoga program of physical postures, breathing exercises, relaxation, and meditation was taught 2 to 3 times a week for 10 weeks (28 yoga sessions total).<br><br>Comparison: PE as usual	- Mood (POMS- Short Form)<br>- Affect (Positive and Negative Affect Schedule for Children)<br>- Stress (Perceived Stress Scale)<br>- Positive psychology (Inventory of Positive Psychological Attitudes)<br>- Resilience (Resilience Scale)<br>- Anger (State Trait Anger Expression Inventory-2TM)<br>- Mindfulness (Child Acceptance Mindfulness Measure)<br><br>*One week before and after.*	RCT	• Small sample size.<br>• Would have been ideal to randomise individually but being in a school setting required allocation at the classroom level<br>• Moderate attendance at the yoga classes
Staiano et al (2013)[45]<br>*USA*	N = 54	Overweight and obese students from an urban public high school.<br>Gender: 55.6%	All exergame participants were encouraged to play the Nintendo Wii Active game for 30-60 minutes per school day in a lunch-time or after-school program. Cooperative EG	- Self-efficacy (Exercise Confidence Survey)<br>- Self-esteem (Rosenberg Self-Esteem scale)<br>- Peer support (Friendship Quality Questionnaire)<br><br>*Baseline, T2 (10 weeks), T3 (20 weeks)*	RCT	• Sample bias: small sample from one school and some attrition

For peer review only - http://bmjopen.bmj.com/site/about/guidelines.xhtml

		female<br>Age: 15-19<br>Ethnicity: African American	participants worked with a peer to expend calories and earn points together, whereas competitive EG participants competed against a peer.<br><br>Comparison: regular daily activities			

**Grey Literature**					
**Authors (Date) *Country***	**Participant description**	***Project/organisation* Type of intervention**	**Evaluation aims and objectives**	**Study design**	**Limitations**
Potter and Stubbs (2015)[49] *UK*	N = 1498 participated in in-school workshops<br>N = 2096 in the final sharing events.<br>Age: 11-13<br>Participants are from urban and rural areas of deprivation	*DanceQuest* – watching and performing dance	1.Examine the processes, outcomes and impacts for both individuals and organisations participating in DanceQuest 2014/15<br>-Measure the successes of DanceQuest 2014/15 against the prescribed aims and objectives established at the outset<br>-Investigate the longer-term impacts of DanceQuest 2012/2015 described and presented through representative case studies<br>-Draw out any general lessons for effective practices for future, similar projects delivered by Children & the Arts.	Qualitative – interviews, observations and photographs throughout	• Focus on the positive WB outcomes<br>• Face value reporting used
BOP Consulting (2016)[48] *UK*	N= 23<br>Age: 8 - 21<br>London (UK) Boroughs of Tottenham and Haringey	*Jackson Lane* - Multi-arts venue with a programme of contemporary circus, comedy, dance and performance.	Assess the impact of the programme:<br>1. Who is reached by Jacksons Lane's programmes?<br>2. What was participants' experience of them?<br>3. What difference did participating make?	Qualitative – semi-structured interviews with participants and volunteers	• Focus on the positive WB outcomes<br>• Face value reporting used
Mansfield et al	Population target: inactive people in the London Borough of	*Health and Sport Engagement (HASE) Project* – sport	Conduct a longitudinal process evaluation examining the key ingredients of successful	Qualitative - focus groups,	• attempted to search for disconfirming cases and consider

For peer review only - http://bmjopen.bmj.com/site/about/guidelines.xhtml

(2016)[47] *UK*	Hounslow		HASE community programmes and identify challenges in designing, delivering and evaluating the HASE projects.	structured observations, in-depth interview methods	the negative wellbeing impact of sport participation

For peer review only

For peer review only - http://bmjopen.bmj.com/site/about/guidelines.xhtml

Table 4 Summary of numerical results of included studies

Author (date)	Outcome (measure)	Baseline		Follow up 1		Follow up 2	
		Intervention Numbers Mean (SD)	Control Numbers Mean (SD)	Intervention Numbers Mean (SD)	Control Numbers Mean (SD)	Intervention Numbers Mean (SD)	Control Numbers Mean (SD)
Akandere and Demir (2011)[39]	Depression (Beck Depression Scale)	N =60<br>15.72 (7.004)	N= 60<br>16.53 (5.922)	N= 60<br>13.90 (5.568)*#	N= 60<br>17.48 (7.740)[X]	N/A	N/A
Amorose et al (2009)[46]	-Need satisfaction; sport competence, need for autonomy, need for relatedeness.	N=93<br>Sport competence: 5.71 (0.84)<br>Need for autonomy: 3.79 (0.79)<br>Need for relatedness: 5.47 (1.15)		N=93<br>Sport competence: 5.50 (1.07)<br>Need for autonomy: 3.76 (0.59)<br>Need for relatedness: 5.50 (1.21)		N/A	N/A
	-Self-esteem (10 item Rosenberg's Self Esteem Scale)	N=93<br>3.21 (0.45)		N=93<br>3.21 (0.47)			
	-Burnout (15 item Athlete Burnout Questionnaire)	N=93<br>2.05 (0.71)		N=93<br>2.15 (0.64)			
Kanojia et al (2013)[40]	Anger (16 item questionnaire)	N=25<br>Postmenstrual phase: Initial cycle 8.84 (4.01), Premenstrual phase: Initial cycle 15.0(5.92)▪	N=25<br>Postmenstrual phase: Initial cycle 9.12(4.41), Premenstrual phase: Initial cycle 14.32(5.24)▪	N= NR<br>Postmenstrual 2nd cycle 7.76 (3.53)*, Premenstrual 2nd cycle 9.52 (4.70)*▪	N= NR<br>Postmenstrual 2nd cycle 9.04(4.33), Premenstrual 2nd cycle 14.28(4.89)▪	N= NR<br>Postmenstrual 3rd cycle 7.92 (4.29) Premenstrual 3rd cycle 8.52 (4.15)*+	N= NR<br>Postmenstrual 3rd cycle 8.96(4.65) Premenstrual 3rd cycle 13.12(4.83)▪

For peer review only

For peer review only - http://bmjopen.bmj.com/site/about/guidelines.xhtml

Author (date)	Outcome (measure)	Baseline		Follow up 1		Follow up 2	
		Intervention Numbers Mean (SD)	Control Numbers Mean (SD)	Intervention Numbers Mean (SD)	Control Numbers Mean (SD)	Intervention Numbers Mean (SD)	Control Numbers Mean (SD)
	Trait anxiety (40 Item questionnaire)	N=25 Postmenstrual phase: Initial cycle 40.64 (6.22), Premenstrual phase: Initial cycle 46.96 (5.87)▪	N=25 Postmenstrual phase: Initial cycle 41.6(5.49), Premenstrual phase: Initial cycle 46.76(5.33)▪	N=NR Postmenstrual 2nd cycle 39.40 (6.69), Premenstrual 2nd cycle 41.48 (5.77)*▪	N= NR Postmenstrual 2nd cycle 40.24(6.97), Premenstrual 2nd cycle 45.80(6.41)▪	N= NR Postmenstrual 3rd cycle 37.24 (9.14)*+ Premenstrual 3rd cycle 40.80 (5.75)*▪	N= NR Postmenstrual 3rd cycle 38.64 (12.76) Premenstrual 3rd cycle 43.88(7.06)
	Depression (10 item questionnaire)	N=25 Postmenstrual phase: Initial cycle 6.84 (3.10) Premenstrual phase: Initial cycle 10.72 (4.19)▪	N=25 Postmenstrual phase: Initial cycle 6.36(4.13), Premenstrual phase: Initial cycle 9.72(3.89)▪	N= NR Postmenstrual 2nd cycle 3.96 (2.59)* Premenstrual 2nd cycle 5.92(3.76)*▪	N= NR Postmenstrual 2nd cycle 6.24(4.98), Premenstrual 2nd cycle 9.56(3.22)▪,	N= NR Postmenstrual 3rd cycle 3.12 (2.71)*+ Premenstrual 3rd cycle 4.76(2.82)*+▪	N= NR Postmenstrual 3rd cycle 6.07(2.81) Premenstrual 3rd cycle 9.36(2.96)▪
	Subjective wellbeing (50 item questionnaire)	N=25 Postmenstrual phase: Initial cycle 41.72 (16.05), Premenstrual phase: Initial cycle 53.92 (20.35)▪	N=25 Postmenstrual phase: Initial cycle 45.6(14.05), Premenstrual phase: Initial cycle 51.04(14.89)	N= NR Postmenstrual 2nd cycle 39.64(16.07)*, Premenstrual 2nd cycle 44.48 (17.87)*▪	N= NR Postmenstrual 2nd cycle 44.68(16.5), Premenstrual 2nd cycle 50.40(18.67),	N= NR Postmenstrual 3rd cycle 37.20(15.17)*+ Premenstrual 3rd cycle 40.24 (16.22)*+	N= NR Postmenstrual 3rd cycle 43.96(14.01) Premenstrual 3rd cycle 49.76(17.02)▪
Kim and Kim (2007)[41]	Positive wellbeing (Subjective Exercise Experiences Scale)	Ice skating (n=84): 19 (3.9) Hip-hop dance (n=45): 16.3 (4.2) Body conditioning (n=64): 15.3 (2.9) Aerobic dance (n=84): 16.8 (4.0)		Ice skating (n=84): 20.4 (3.4)[X] Hip-hop dance (n=45): 19.7 (3.4)* Body conditioning (n=64): 18 (2.8)[X] Aerobic dance (n=84): 19.9 (3.9)*		n/a	n/a
	Psychological distress (Subjective Exercise Experiences Scale)	Ice skating: 8.3 (3.9) Hip-hop dance: 9.8 (4.6) Body conditioning: 10.7 (4.1) Aerobic dance: 9.4 (4.2)		Ice skating: 8.1 (3.9)[X] Hip-hop dance: 7.3 (4.2)* Body conditioning: 9.6 (3.2)[X] Aerobic dance: 6.7 (2.9)*		n/a	n/a

For peer review only - http://bmjopen.bmj.com/site/about/guidelines.xhtml

Author (date)	Outcome (measure)	Baseline		Follow up 1		Follow up 2	
		Intervention Numbers Mean (SD)	Control Numbers Mean (SD)	Intervention Numbers Mean (SD)	Control Numbers Mean (SD)	Intervention Numbers Mean (SD)	Control Numbers Mean (SD)
	Fatigue (Subjective Exercise Experiences Scale)	Ice skating: 10.9 (5.4)<br>Hip-hop dance: 16.2 (4.4)<br>Body conditioning: 15.9 (4.4)<br>Aerobic dance: 14.4 (5.0)		Ice skating: 13.9 (5.3)[X]<br>Hip-hop dance: 12.9 (4.7)*<br>Body conditioning: 13.9 (4.1)[X]<br>Aerobic dance: 11.2 (4.3)*		n/a	n/a
Li et al (2015)[42]	Self-esteem (SES)	N=101<br>31.17 (3.69)	N=105<br>31.41 (3.29)	N= 96 (101 included in ITT analysis)<br>31.56 (3.30)	N= 105 (105 included in ITT analysis) 31.31(3.27)	N= 93 (ITT analysis)<br>30.81 (3.45)	N= 101 (ITT analysis)<br>31.0 (3.71)
	Mood / mindfulness (POMS scale)	N=101<br>102.3 (16.14)	N=105<br>103.5 (17.34)	N= 96 (101 included in ITT analysis)<br>106 (15.68)	N= 105 (105 included in ITT analysis) 107.4 (17.95)	N= 93 (ITT analysis)<br>103.8 (16.78)	N= 101 (ITT analysis)<br>104.6 (16.89)
	QoL (WHOQOL-BREF)	N=101<br>55.84 (6.65)	N=105<br>54.94 (6.45)	N= 96 (101 included in ITT analysis)<br>55.09 (6.93)	N= 105 (105 included in ITT analysis) 54.26(7.02)	N= 93 (ITT analysis)<br>56.29 (7.45)	N= 101 (ITT analysis)<br>55.61 (7.45)
	Attention (Schulte Grid)	N=101<br>213.9 (58.84)	N=105<br>224.6 (47.52)	N= 96 (101 included in ITT analysis)<br>192.4 (47.14)	N= 105 (105 included in ITT analysis)<br>210.4 (54.15)[#]	N= 93 (ITT analysis)<br>193.9 (54.31)	N= 101 (ITT analysis)<br>202.8 (58.34)
	Stress (CPSS Scale)	N=101<br>24.22 (5.18)	N=105<br>23.91 (5.50)	N= 96 (101 included in ITT analysis)<br>23.53 (5.40)	N= 105 (105 included in ITT analysis)<br>22.60 (5.43)	N= 93 (ITT analysis)<br>22.72 (5.72)	N= 101 (ITT analysis)<br>23.22 (5.72)
	Self-symptom intensity (SCL-90 scale)	N=101<br>142.9 (33.58)	N=105<br>142.1(32.77)	N= 96 (101 included in ITT analysis)<br>135.6 (31.3)	N= 105 (105 included in ITT analysis)<br>136.2 (32.4)	N= 93 (ITT analysis)<br>130.6(34.83)	N= 101 (ITT analysis)<br>130.4(31.94)
Lindgren et al (201)[43]	General self-efficacy (GSES)	N= 55<br>Median (IQR)<br>32.0 (11.0-54.0)	N= 53<br>Median (IQR)<br>32.0 (14.0-47.0)	N= 27<br>Median (IQR)<br>28.0 (15.0–48.0)*[#]	N= 36<br>Median (IQR)<br>35.0 (16.0–48.00)[X]	n/a	n/a
	Specific self-efficacy (SPBESQ)	N= 56<br>Median (IQR)<br>Support: 9.0 (3.0-18.0)	N= 54<br>Median (IQR)<br>Support: 8.0 (3.0–	N= 27<br>Median (IQR)<br>Support: 8.0 (3.0–17.0)[X]	N= 36<br>Median (IQR)<br>Support: 7.0 (3.0–18.0)[X]		

For peer review only - http://bmjopen.bmj.com/site/about/guidelines.xhtml

Author (date)	Outcome (measure)	Baseline		Follow up 1		Follow up 2	
		Intervention Numbers Mean (SD)	Control Numbers Mean (SD)	Intervention Numbers Mean (SD)	Control Numbers Mean (SD)	Intervention Numbers Mean (SD)	Control Numbers Mean (SD)
		Social: 22.0 (7.0-35.0)	16.0)<br>Social: 18.5 (7.0–37.0)	Social: 19.0 (7.0–36.0)[X]	Social: 19.0 (8.0–31.0)[X]		
Noggle et al (2012)[44]	Mood (POMS-SF)	N=36<br>Mood Disturbance (-):42.8 (19.3)<br>Tension anxiety (-):6.4 (4.7)<br>Depression-dejection (-):5.1 (5.0)<br>Anger Hostility (-):6.5 (4.7)<br>Vigor activity (+):9.8 (4.4)<br>Fatigue inertia (-): 8.3 (5.7)<br>Confusion bewilderment (-): 6.8 (3.5)	N=15<br>Mood Disturbance (-):44.5 (10.2)<br>Tension anxiety (-):6.7 (2.8)<br>Depression-dejection (-):4.9 (3.0)<br>Anger Hostility (-):6.3 (2.7)<br>Vigor activity (+):10.2 (3.8)<br>Fatigue inertia (-):9.8 (4.5)<br>Confusion bewilderment (-): 6.6 (2.7)	N=35<br>Mood Disturbance (-):38.4 (16.9)[#] medium-large effect size = 0.689 [Cohen's d]<br>Tension anxiety (-):5.1 (3.6)[#] Large effect size = 0.870 [Cohen's d]<br>Depression-dejection (-):4.7 (4.9)<br>Anger Hostility (-): 5.7 (5.0)<br>Vigor activity (+):9.3 (4.0)<br>Fatigue inertia (-): 7.2 (5.2)<br>Confusion bewilderment (-): 6.3 (3.5)	N=15<br>Mood Disturbance (-):51.2 (20.1)<br>Tension anxiety (-):9.3 (5.8)<br>Depression-dejection (-):6.3 (4.2)<br>Anger Hostility (-):7.1 (4.5)<br>Vigor activity (+):10.9 (3.5)<br>Fatigue inertia (-):9.3 (4.6)<br>Confusion bewilderment (-): 8.3 (4.1)	n/a	n/a
	Stress (Perceived Stress Scale)	N=36<br>19.2 (7.4)	N=15<br>19.1 (3.8)	N=35<br>18.6 (6.2)	N=15<br>20.3 (5.4)	n/a	n/a

For peer review only - http://bmjopen.bmj.com/site/about/guidelines.xhtml

Author (date)	Outcome (measure)	Baseline		Follow up 1		Follow up 2	
		Intervention Numbers Mean (SD)	Control Numbers Mean (SD)	Intervention Numbers Mean (SD)	Control Numbers Mean (SD)	Intervention Numbers Mean (SD)	Control Numbers Mean (SD)
	Positive psychology (Inventory of Positive Psychological Attitudes)	N=36<br>Positive psych attributes (+):4.5 (1.0)<br>Life Purpose/satisfaction (+):4.7 (1.0)<br>Self conf during stress (+): 4.2 (1.0)	N=15<br>Positive psych attributes (+):4.5 (0.78)<br>Life Purpose/satisfaction (+):4.8 (0.94)<br>Self conf during stress (+): 4.2 (0.67)	N=35<br>Positive psych attributes (+):4.5 (1.2)<br>Life Purpose/satisfaction (+):4.8 (1.1)<br>Self conf during stress (+): 4.3 (0.98)	N=15<br>Positive psych attributes (+):4.2 (0.88)<br>Life Purpose/satisfaction (+):4.6 (0.88)<br>Self conf during stress (+): 4.0 (0.90)	n/a	n/a
	Resilience (Resilience Scale)	N=36<br>132.9 (18.4)	N=15<br>132.1 (12.4)	N=35<br>131.9 (24.5)	N=15<br>127.9 (23.4)	n/a	n/a
	Affect (Positive and Negative Affect Schedule for Children)	N=36<br>Positive affect (+):50.1 (11.5)<br>Negative affect (-): 32.1 (12.5)	N=15<br>Positive affect (+):47.7 (9.4)<br>Negative affect (-): 28.8 (7.7)	N=35<br>Positive affect (+):48.6 (11.7)<br>Negative affect (-): 29.4 (11.5)[#] Medium-large effect size = 0.659 [Cohen's d]	N=15<br>Positive affect (+):49.2 (11.3)<br>Negative affect (-): 38.4 (15.5)	n/a	n/a
	Mindfulness (Child Acceptance Mindfulness Measure)	N=36<br>53.9 (8.6)	N=15<br>52.3 (6.7)	N=35<br>53.4 (7.8)	N=15<br>49.4 (7.2)	n/a	n/a
	Anger (State Trait Anger Expression Inventory-2TM)	N=36<br>Inward (-): 16.4 (4.2)<br>Outward (-): 17.2 (5.7)<br>Control (+): 22.8 (5.5)	N=15<br>Inward (-): 15.9 (3.3)<br>Outward (-): 16.5 (4.0)<br>Control (+):22.7 (5.3)	N=35<br>Inward (-): 16.8 (4.9)<br>Outward (-): 16.9 (5.5)<br>Control (+): 22.4 (6.1)	N=15<br>Inward (-): 17.9 (4.6)<br>Outward (-): 17.1 (3.7)<br>Control (+): 20.9 (3.7)	n/a	n/a

For peer review only - http://bmjopen.bmj.com/site/about/guidelines.xhtml

Author (date)	Outcome (measure)	Baseline		Follow up 1		Follow up 2	
		Intervention Numbers Mean (SD)	Control Numbers Mean (SD)	Intervention Numbers Mean (SD)	Control Numbers Mean (SD)	Intervention Numbers Mean (SD)	Control Numbers Mean (SD)
Staiano et al (2013)[45]	Self-efficacy (Exercise Confidence Survey)	Cooperative (n = 19): 38.16 (12.12) Competitive (n = 19): 36.37 (13.97)	n = 16 37.38 (12.07)	Cooperative (n = 18): 42.11 (13.58) Competitive (n = 17): 37.65 (10.03)	n = 14 34.57 (11.75)	Cooperative (n = 14): 43.29 (13.40) Competitive (n = 11): 38.82 (8.82)	n = 10 35.30 (8.76)
	Self-esteem (Rosenberg Self-Esteem scale)	Cooperative (n = 19): 22.79 (4.45) Competitive (n = 19): 23.74 (6.47)	N=16 22.69 (3.96)	Cooperative (n = 18): 22.67 (5.91) Competitive (n = 18): 23.11 (4.78)	N=15 22.40 (5.38)	Cooperative (n = 13): 24.08 (3.88) Competitive (n = 9): 22.33 (5.74)	N=11 20.45 (5.82)
	Peer support (Friendship Quality Questionnaire)	Cooperative (n=19): 71.89 (12.43) Competitive (n=19): 64.37 (19.58)	N=16 70.13 (18.16)	Cooperative (n = 18): 75.22 (13.39) Competitive (n = 18): 72.44 (10.78)	N=15 72.33 (17.15)	Cooperative (n = 11): 80.18 (8.59) Competitive (n = 13): 76.92 (14.04)	N=10 59.70 (20.67)

Key
*p<0.05 from baseline to follow up within groups, ^{X} p>0.05 from baseline to follow up within groups, $^{\#}$p<0.05 between groups at follow up
Kanojia et al (2013): *p<0.05 in comparison with initial cycle, +p<0.05 in comparison with 2nd cycle, ▪ p<0.05 comparison between pre- and postmenstrual phase
NR = not reported

For peer review only - http://bmjopen.bmj.com/site/about/guidelines.xhtml

RESULTS

Search results

After the removal of duplicates the electronic searches returned 5597 records for title and abstract screening. Of these, 143 relevant articles remained for full text assessment as well as 60 additional texts identified through other sources (six through hand searching the reference lists of included reviews, and 54 grey literature submissions were found: 12 received through the call for evidence, 33 via the extended search for grey literature and 9 PhDs found on Ethos). After screening the 203 full texts, eleven studies were included in the systematic review. The search screening process is illustrated in Figure 1.

Study characteristics

The systematic review includes seven randomised control trials (RCTs)[39-45] (with a total of 884 participants) and one cohort study[46] (93 participants) from the published literature. Three evaluation reports were included from the grey literature. A summary of the characteristics (country, number and description of participants, intervention and comparison, outcomes and measures/aims and objective, study design, and limitations) of the included papers is presented in Table 3. Table 4 provides a summary of the numerical results for each published study (including number of participants, mean scores [SD] for each outcome measure at each measurement point, and a summary of the results).

The included studies investigated the effects of a range of sport and dance interventions; the most common form of intervention reported were based on meditative practices including yoga[40,44] and Baduanjin Qigong.[42] Other interventions reported included body conditioning, aerobic exercise,[41] dance forms delivered through dance training,[39] hip-hop dance,[41] an empowerment-based exercise intervention programme[43] and specifically identified sports including aerobic exercise, body conditioning, hip-hop dancing, and ice skating[41] and Nintendo Wii Active Games.[45] Descriptions of interventions tended to be superficial including brief comment

For peer review only - http://bmjopen.bmj.com/site/about/guidelines.xhtml

about the frequency and type of activity, the qualifications of the instructor and the delivery site. The cohort study followed volleyball players through a season of competitive games.[46] Interventions in six of the RCT studies and in the cohort study were led by sport or dance instructors in formal group sessions. One RCT used the Nintendo Wii Active Games Programme incorporating a cooperative or competitive peer-to-peer method of participation. A wide range of wellbeing measures were used and are summarised in appendix 5.

Projects reported in the grey literature included the following interventions: martial arts, dance, gym-based exercise, exercise classes, swimming, netball, cycling and football,[47] circus-based skills (e.g. juggling, balancing, diabolo),[48] and a range of dance forms.[49] Interventions were led by instructors in group settings. Wellbeing was evaluated using descriptive statistics and/or thematic analysis from surveys, focus groups, interviews, and structured observations.

All of the included studies were carried out in countries categorised in the same group as the UK in the OECD Development Assistance Committee categories apart from two (one took place in India,[40] and the other was based in Korea[41]). The sample participants in these two studies were conducted with University students and likely to be relative high in socioeconomic status and so were included.

INSERT HERE Figure 1 PRISMA flow diagram of the search screening process

For peer review only - http://bmjopen.bmj.com/site/about/guidelines.xhtml

For peer review only

Study quality

The scores for the included studies from the What Works Centre for Wellbeing quality checklist for quantitative data is presented in table 2. The most frequent methodological weaknesses within the studies (with four or fewer studies meeting the criteria) were the absence of an intent-to-treat design, not having a clear process for determining and reporting drop-out and dose, not having an appropriate method for the treatment of missing data, not controlling for confounding factors, not being able to blind participants or measurements, and not including assessment information independent of the participants. Common (all studies meeting the criteria) strengths included; using appropriate measures, independent of treatment measures, giving measures before and after the intervention/control, and using appropriate methods to analyse the data. The results of the quality checklist varied across studies, with Amorose et al. (2009)[46] scoring the worst (9 criteria met) and Li et al. (2015)[42] scoring the highest (21 criteria met).

The use of the GRADE schema for judging quality of evidence means that despite the predominance of RCT designs, overall the quality of the published evidence on sport and dance interventions to enhance wellbeing is low in respect of there being very little evidence in total, methodological limitations noted above, small sample sizes in studies and some sample bias.

Using the PHE Arts for Health and Wellbeing Evaluation Framework, the evidence from the grey literature were judged to have a high degree of credibility. The strongest reports included descriptive and theoretical detail about evaluation methods and acknowledged the limitations of evaluation design. Two studies reported both pre-project and post-project data. It was not always clear how themes were identified and developed and it was not always apparent that conclusions emerged from comprehensive data treatment. One report made a clear attempt to search for disconfirming cases and consider the negative wellbeing impact of sport participation[47] but evaluation reports tended to focus only on the positive impacts of sport and dance. Further, there was a tendency in evaluations on dance and performance to rely on face value reporting of

For peer review only - http://bmjopen.bmj.com/site/about/guidelines.xhtml

participants' accounts rather than developing latent forms of thematic analysis informed by identified theory where appropriate.

The effect of meditative sport activity on wellbeing

Three published RCT studies assessed the effectiveness of meditative practices including yoga[40,44] and Baduanjin-Qigong[42] on wellbeing in young people. All three studies used several different measures of wellbeing including mood scales for rating anger, anxiety, positive and negative affect, confusion/bewilderment, and stress, anxiety and depression.[40, 42 ,44] One study also included measures of self-esteem, quality of life, mindfulness and resilience. [42] Two studies reported significantly improved wellbeing outcomes from taking part in yoga compared to a control group.[40, 44] One study found significant reductions between groups in total mood disturbance (medium-large effect size = 0.689 [Cohen's d], p=0.015), tension and anxiety (large effect size = 0.870 [Cohen's d], p=0.002) and negative affect (medium-large effect size = 0.659 [Cohen's d], p=0.006).[44] The second study found a significant reduction at 3 months compared to baseline in self-reported depression (effect size=not reported [nr], postmenstrual phase p<0.001, premenstrual phase p<0.001), anxiety (effect size=nr, postmenstrual p<0.05 premenstrual p<0.001), and anger (effect size=nr, premenstrual p<0.001), as well as an improved overall sense of wellbeing (effect size=nr, postmenstrual p<0.001, premenstrual p<0.001).[40] One study reported no significant difference in self-esteem, mindfulness, quality of life, stress or 'sympton' intensity in young people taking part in Baduanjin-Qigong compared to usual exercise practice.[42] No grey literature on yoga and wellbeing was included in this review.

The effect of group / team sport on wellbeing

Two published RCT studies [43,45] and one cohort study[46] examined the wellbeing outcomes of group sport activities. Two of these studies measured wellbeing using self-efficacy scales.[43, 45] Two studies included a measure of self-esteem.[45, 46] One study used a friendship quality assessment as a measure of wellbeing.[45] One study measured wellbeing outcomes relating to need satisfaction theory (competence, autonomy and relatedness);[46] an established approach to

For peer review only - http://bmjopen.bmj.com/site/about/guidelines.xhtml

personal wellbeing research in sport psychology. The two studies using self-efficacy measures reported statistically significantly improved scores after taking part in group sport interventions comparted to the control (effect size=nr, p=0.037;[43] cooperative condition [M=43.29, SD=13.40] vs control group [M=35.30, SD=8.76], t=2.99, p=0.005).[45]

Both these studies employed interventions that were tailored to the needs of the participants and included elements of peer support. Significant increases in friendship quality were reported in taking part in sport compared to no sport (control condition: M=59.70 SD=20.67; cooperative condition: M=80.18, SD-8.59, t=2.76, p=0.010; competitive condition: M=76.92, SD=14.04, t=3.66, p=.001).[45] No significant differences were reported for self-esteem scores between sport intervention groups compared to control.[45] Changes in sports players' need to feel competent, autonomous and connected to others over the course of a sporting season were found to be positively related to changes in their overall sense of self-esteem.[46] Qualitative findings from the one grey literature report identified negative and positive aspects of wellbeing associated with engagement in community sport including enhanced feelings of social connectedness, pleasure and sense of purpose as well as concerns related to personal capability, competence and unfavourable comparisons to peers who are 'sporty'. [47]

The effect of group dance on wellbeing

Two published RCT studies examined the wellbeing outcomes (mood, fatigue scores and levels of depression) of group dance activities.[39,41] One used a bespoke dance training programme,[39] the other compared dance activities with sport and fitness activities.[41] Taking part in dance exercise to music (aerobics) and hip hop dancing aerobics were reported to significantly improve self-reported positive wellbeing and reduce distress and fatigue at the end of the intervention (effect size=nr, p<0.05).[41] Significant improvements on the self-reported Beck Depression Scale (0-9 = not depressed; 10-15 = low depression; 16-23 = medium level depression, 24+ = depressive) in participants not diagnosed with depression were reported from a dance training intervention (M=

For peer review only

13.90, SD=5.568) compared to control (M=17.48, SD=7.740); t=2.911, p=0.004.[39] The grey literature reported questionnaire and interview results showing positive wellbeing associations from dance interventions in terms of increased confidence, sense of purpose and fun and exhilaration. [48, 49] Dance was also found to enhance, happiness, relaxation, playfulness, fun, social connectedness, aspiration, ambition and reduce isolation.[48]

DISCUSSION

Summary of evidence

To our knowledge this is the first systematic review on sport and dance interventions in healthy young people (15-24 years) to promote subjective wellbeing. Overall, the published evidence suggests that meditative physical activity (yoga and Baduanjin Qigong) has the potential to improve subjective wellbeing in terms of reduced anxiety, depression and anger, and enhanced positive mood in young people. The evidence also shows that taking part in dance can lead to positive wellbeing outcomes of in terms of mood enhancement and self-reported reductions in feelings of depression in some youth populations. Group-based and peer supported delivery mechanisms in sport and dance programmes may support wellbeing enhancement for young people. Unpublished grey literature illustrated that taking part in or watching dance, or other forms of performance-based physical activity and community sport instil positive wellbeing feelings such as exhilaration and sense of purpose, and increased confidence, self-esteem and feelings of belonging and purpose. Taking part in community sport was also associated with negative wellbeing connected to concerns about competency and capability. The findings should be treated with caution because the quality of the published evidence on sport and dance interventions to enhance wellbeing is judged to be low. The evidence is sparse and there are methodological limitations including a lack of rigour in research design and conduct, small sample sizes in studies and some sample bias.

For peer review only - http://bmjopen.bmj.com/site/about/guidelines.xhtml

For peer review only

Strengths and limitations of the review

Strengths

The comprehensive search strategy ensures that this review represents a complete summary of all existing eligible studies published prior to September 2016, and the pre-publication of our protocol on PROSPERO ensures methodological transparency and mitigates against potential post-hoc decision making which can introduce bias to the process. Dual screening of searches and data extraction and independent quality assessment of included reviews ensured a rigorous process. We employed broad and inclusive criteria for wellbeing outcome measures to make the results more relevant to sport and dance policy and service provision. Including grey literature of recent unpublished data (completed 2013-2016) from evaluations reduced the potential risk of publication lag, wherein possible important new evidence that has not yet been included in published reports is not identified and included.

Limitations

There was a wide variety of wellbeing measures used in the studies including variation in measures used for the same named wellbeing outcome. A great deal of heterogeneity across studies meant a meta-analysis was not appropriate in this systematic review. The focus on a specific target age group will have excluded evidence from studies that have aggregated data across younger and older age groups in their analysis. The use of the GRADE criteria introduces an element of subjective judgement although it attempts to reduce subjective bias by introducing a rigorous process of quality assessment. A consistent approach to judgements across the outcome (wellbeing) has been applied but it should be recognised that these judgements are open to interpretation.

Implications for policymakers and future research

The evidence in this review is sparse and we know very little about the effect of sport and dance interventions which have the potential to influence the wellbeing of large numbers of people. No published UK studies were eligible for inclusion in this review. It is not possible to conclude that

For peer review only - http://bmjopen.bmj.com/site/about/guidelines.xhtml

For peer review only

findings in this review are generalizable across countries or regionally in the UK. The lack of evidence identified in this review does not necessarily mean that wellbeing benefits are not accrued from taking part in sport and dance. Large scale community sport and dance interventions have the potential to influence the wellbeing of people at population level. Recent national sport strategy in the UK[4, 5] identifies wellbeing as an outcome for sport and physical activity and needs to be accompanied by agreement about definitions and measures of wellbeing, a focus on measuring wellbeing outcomes and an emphasis on evaluating what works to enhance wellbeing in sport and dance. National agencies across the sport, culture, and health sectors (e.g. DCMS, ACE, Sport England, PHE) may be influential in promoting this approach; conversely, a lack of national lead may discourage academic and service delivery stakeholders from prioritising this.

Based on the evidence in this study it is necessary to build evidence on wellbeing outcomes of sport and dance in healthy young people using agreed measures of wellbeing. There is a need for more well designed, rigorous studies which are underpinned by relevant theory. Large-scale randomised controlled designs should be implemented in this target age group. Other rigorous and systematic study designs including evaluation of the complex community context and mechanisms of intervention effectiveness should be considered. The development of a multilevel programme of wellbeing evaluation training would support key policy and service delivery personnel and researchers in the sport and dance sectors in ensuring rigorous evaluation of interventions.

CONCLUSION

The evidence overall for the subjective wellbeing benefits of sport and dance interventions for healthy young people is limited, very selective, and drawn from varied national and cultural contexts. The current state of the evidence means that it is not possible to identify a common effect of sport and dance on the subjective wellbeing of young healthy people or be certain about the influence of such physical activity on peoples' wellbeing. There are large gaps in our knowledge about the effect of sport and dance on the wellbeing of young people. Knowledge should be improved through rigorous complex community intervention research incorporating valid

For peer review only - http://bmjopen.bmj.com/site/about/guidelines.xhtml
For peer review only

comparator groups to determine which sport and dance interventions are most effective in improving wellbeing in young people. Measurement of quantitative outcomes and evaluation of qualitative processes to determine how such interventions achieve their outcomes is needed.

FUNDING

This work was supported by ESRC research grant (number ES/NOO3721/1) which forms part of the evidence review programme within the UK What Works Centre for Wellbeing.

CONTRIBUTOR SHIP STATEMENT

The review was conceived and designed, and the protocol developed by TK, CM, LGD, JL, AJ, ND, PD, ST, GJ, AP, AT AND CV; article screening was carried out by LM, TK, AJ, LGD, JL, CV; data extraction, quality checks data interpretation were completed by LM, TK, AJ, LGD, JL, CV; and the manuscript was drafted by LM and critically reviewed by TK, CM, LGD, JL, AJ, ND, PD, ST, GJ, AP, AT AND CV.

ACKNOWLEDGEMENTS

We acknowledge the expert support for our searches provided by library-based information scientists at Brunel University London and the University of Brighton

DECLARATION OF INTEREST

We have read and understood BMJ policy on declaration of interests and declare that we have no competing interests.

DATA SHARING STATEMENT

The appendix is available as online supplementary material and includes; Appendix 1, demonstration OVID MEDLINE search strategy; Appendix 2, table of excluded studies; Appendix 3, the standardised data extraction form; Appendix 4, the What Works Centre for Wellbeing quality checklist (quantitative studies); Appendix 5, summary of SWB measures used in included studies.

FIGURE LEGEND

Figure 2 PRISMA flow diagram of the search screening process

For peer review only - http://bmjopen.bmj.com/site/about/guidelines.xhtml

For peer review only

REFERENCES

1. Huppert, F.A. and So, T.T., 2013. Flourishing across Europe: Application of a new conceptual framework for defining well-being. *Social Indicators Research*, *110*(3), pp.837-861.

2. Berry C. Wellbeing in four policy areas. *Report by the All-Party Parliamentary Group on wellbeing economics*. Report, New Economics Foundation, London, September 2014.

3. O'Donnell G, Deaton A, Durand M, et al. *Wellbeing and Policy*. London: Legatum Institute, 2014.

4. Department of Culture Media amd Sport.*Sporting Future: a new strategy for an active nation.* London : Crown Copyright; 2015.

5. Sport England.*Towards an active nation strategy 2016-2021.* London : DCMS; 2016.

6. OneDanceUK (2017) About Us. Website

7. Start Active, Stay Active. A report on physical activity from the four home countries'. Chief Medical Officers. 2011 Jul. View PDF

8. Dolan, P. (2014). *Happiness by Design: Change What You Do, Not How You Think*. London:Penguin.

9. Tinkler, L. and Hicks, S., 2011. Measuring subjective well-being. *London: Office for National Statistics*, pp.443-455.

10. Dolan P and Metcalf R. Measuring subjective wellbeing: Recommendations on measures for use by national governments. *Journal of Social Policy* 2012; 41(2): 409-427.

11. Fujiwara D, Kudrna L and Dolan P. *Quantifying and valuing the wellbeing impacts of culture and sport*. London, DCMS https://www.gov.uk/government/publications/quantifying-and-valuing-the-wellbeing-impacts-of-culture-and-sport (2014, accessed 7 April 2016).

12. Huppert, F.A., 2017. Challenges in Defining and Measuring Well-Being and Their Implications for Policy. In *Future Directions in Well-Being* (pp. 163-167). Springer International Publishing.

13. Huppert, F.A. 2017. Measurement really matters. *Measuring Wellbeing Series; discussion paper 2.* What Works Centre for Wellbeing. View PDF

14. Stewart-Brown S, Janmohamed K. Warwick-Edinburgh mental well-being scale. User guide. Version. 2008 Jun;1.

15. Robins RW, Hendin HM, Trzesniewski KH. Measuring global self-esteem: Construct validation of a single-item measure and the Rosenberg Self-Esteem Scale. Personality and social psychology bulletin. 2001 Feb;27(2):151-61.

16. Curran SL, Andrykowski MA, Studts JL. Short form of the Profile of Mood States (POMS-SF): Psychometric information. Psychological assessment. 1995 Mar;7(1):80.

17. What Works Centre for Wellbeing: credible evidence for better decisions to improve lives https://www.whatworkswellbeing.org/

For peer review only - http://bmjopen.bmj.com/site/about/guidelines.xhtml

18. Daykin N, Mansfield L, Payne A, Kay T, Meads C, D'Innocenzo G, Burnett A, Dolan P, Julier G, Longworth L, Tomlinson A. What works for wellbeing in culture and sport? Report of a DELPHI process to support coproduction and establish principles and parameters of an evidence review. Perspectives in public health. 2017 Sep;137(5):281-8.

19. European Sports Charter. 1992. https://search.coe.int/cm/Pages/result_details.aspx?ObjectID=09000016804c9dbb

20. Cavill N, Richardson D, Foster C. Improving Health Through Participation in Sport: a review of research and practice. BHF Health Promotion Research Groups. Oxford University and Sport England. 2012. View PDF

21. Oja P, Titze S, Bauman A, et al. Health benefits of cycling: a systematic review. Scand J Med Sci Sports. 2011 Aug 1;21(4):496-509. https://doi.org/10.1111/j.1600-0838.2011.01299.x View PDF

22. Oja P, Titze S, Kokko S, et al. Health benefits of different sport disciplines for adults: systematic review of observational and intervention studies with meta-analysis. Br J Sports Med. 2015 Jan 7:bjsports-2014. https://doi.org/10.1136/bjsports-2014-093885 View PDF

23. Mammen G, Faulkner G. Physical activity and the prevention of depression: a systematic review of prospective studies. Am J Prev Med. 2013 Nov 30;45(5):649-57. https://doi.org/10.1016/j.amepre.2013.08.001 View PDF

24. Ströhle A. Physical activity, exercise, depression and anxiety disorders. J Neural Transm. 2009 Jun 1;116(6):777. http://dx.doi.org/10.1007/s00702-008-0092-x View PDF

25. Robertson R, Robertson A, Jepson R, Maxwell M. Walking for depression or depressive symptoms: a systematic review and meta-analysis. Mental Health and Physical Activity. 2012 Jun 30;5(1):66-75.

26. Bradt J, Shim M, Goodill SW. Dance/movement therapy for improving psychological and physical outcomes in cancer patients. The Cochrane Library. 2015 Jan 1. http://dx.doi.org/10.1002/14651858.CD007103.pub3 View PDF

27. Ren J, Xia J. Dance therapy for schizophrenia. The Cochrane Library. 2013 Jan 1. http://dx.doi.org/10.1002/14651858.CD006868.pub3 View PDF

28. Meekums B, Karkou V, Nelson A. Dance movement therapy for depression. Cochrane Database of Systematic Reviews. 2015 Feb 19(2):1-54.

29. Biddle SJ, Asare M. Physical activity and mental health in children and adolescents: a review of reviews. Br J Sports Med. 2011 Aug 1:bjsports90185. http://dx.doi.org/10.1136/bjsports-2011-090185 View PDF

30. Moher D, Liberati A, Tetzlaff J, et al. Preferred reporting items for systematic reviews and meta-analyses: the PRISMA statement. Int J Surg 2010;8:336–41.

31. Burkhardt J, Brennan C. The effects of recreational dance interventions on the health and well-being of children and young people: A systematic review. Arts & Health. 2012 Jun 1;4(2):148-61. http://dx.doi.org/10.1080/17533015.2012.665810

For peer review only - http://bmjopen.bmj.com/site/about/guidelines.xhtml

32. Koch S, Kunz T, Lykou S, Cruz R. Effects of dance movement therapy and dance on health-related psychological outcomes: A meta-analysis. The Arts in Psychotherapy. 2014 Feb 28;41(1):46-64. http://dx.doi.org/10.1016/j.aip.2013.10.004 View PDF

33. Korber K. Quality assessment of economic evaluations of health promotion programs for children and adolescents—a systematic review using the example of physical activity. Health economics review. 2015 Nov 24;5(1):35. http://dx.doi.org/10.1186/s13561-015-0071-5 View PDF

34. Liu M, Wu L, Ming Q. How does physical activity intervention improve self-esteem and self-concept in children and adolescents? Evidence from a meta-analysis. PloS one. 2015 Aug 4;10(8):e0134804. http://dx.doi.org/10.1371/journal.pone.013480 View PDF

35. Rössler R, Donath L, Verhagen E, Junge A, Schweizer T, Faude O. Exercise-based injury prevention in child and adolescent sport: a systematic review and meta-analysis. Sports medicine. 2014 Dec 1;44(12):1733-48. View PDF

36. Thompson Coon J, Boddy K, Stein K, Whear R, Barton J, Depledge MH. Does participating in physical activity in outdoor natural environments have a greater effect on physical and mental wellbeing than physical activity indoors? A systematic review. Environmental science & technology. 2011 Feb 3;45(5):1761-72. http://pubs.acs.org/doi/abs/10.1021/es102947t View PDF

37. Daykin N, Joss T. Public Health England Arts and Health Evaluation Framework. 2016 Jan. View PDF

38. Snape, D., Meads, C., Bagnall, A., Tregaskis, O. and Mansfield, L., 2017. What Works Wellbeing: a guide to our evidence review methods. View PDF

39. Akandere M, Demir B. The effect of dance over depression. Coll Antropol. 2011 Sep 30;35(3):651-6. View PDF

40. Kanojia S, Sharma VK, Gandhi A, Kapoor R, Kukreja A, Subramanian SK. Effect of yoga on autonomic functions and psychological status during both phases of menstrual cycle in young healthy females. Journal of clinical and diagnostic research: JCDR. 2013 Oct;7(10):2133-9. http://dx.doi.org/10.7860/JCDR/2013/6912.3451 View PDF

41. Kim S, Kim J. Mood after various brief exercise and sport modes: aerobics, hip-hop dancing, ice skating, and body conditioning. Perceptual and motor skills. 2007 Jun;104(3_suppl):1265-70. http://dx.doi.org/10.2466/PMS.104.3.1265-1270 View PDF

42. Li M, Fang Q, Li J, et al. The effect of Chinese traditional exercise-Baduanjin on physical and psychological well-being of college students: a randomized controlled trial. PloS one. 2015 Jul 9;10(7):e0130544. https://doi.org/10.1371/journal.pone.0130544 View PDF

43. Lindgren EC, Baigi A, Apitzsch E, Bergh H. Impact of a six-month empowerment-based exercise intervention programme in non-physically active adolescent Swedish girls. Health Education Journal. 2011 Mar;70(1):9-20. https://doi.org/10.1177/0017896910379366 View PDF

44. Noggle JJ, Steiner NJ, Minami T, Khalsa SB. Benefits of yoga for psychosocial well-being in a US high school curriculum: a preliminary randomized controlled trial. J Dev Behav Pediatr. 2012 Apr 1;33(3):193-201. https://doi.org/10.1097/DBP.0b013e31824afdc4 View PDF

45. Staiano AE, Abraham AA, Calvert SL. Adolescent exergame play for weight loss and psychosocial improvement: a controlled physical activity intervention. Obesity. 2013 Mar 1;21(3):598-601. https://doi.org/10.1002/oby.20282 View PDF

For peer review only - http://bmjopen.bmj.com/site/about/guidelines.xhtml

46. Amorose AJ, Anderson-Butcher D, Cooper J. Predicting changes in athletes' well being from changes in need satisfaction over the course of a competitive season. Res Q Exerc Sport. 2009 Jun 1;80(2):386-92. http://dx.doi.org/10.1080/02701367.2009.10599575 View PDF

47. Mansfield L, Kay T, Anokye N, Fox-Rushby J. The Health and Sport Engagement (HASE) intervention and evaluation project: the design, delivery and evaluation of a complex community sport intervention for improving physical activity, health and wellbeing. Volume 1: Evaluation of the Design and Delivery of the HASE project. Forthcoming 2017.

48. BOP Consulting. The Social Impact of Jacksons Lane. 2016 Aug. View PDF

49. Potter S, Stubbs FP. DanceQuest 2012-15 Evaluation Report. 2015.

For peer review only

For peer review only - http://bmjopen.bmj.com/site/about/guidelines.xhtml

Figure 1 PRISMA flow diagram of the search screening process

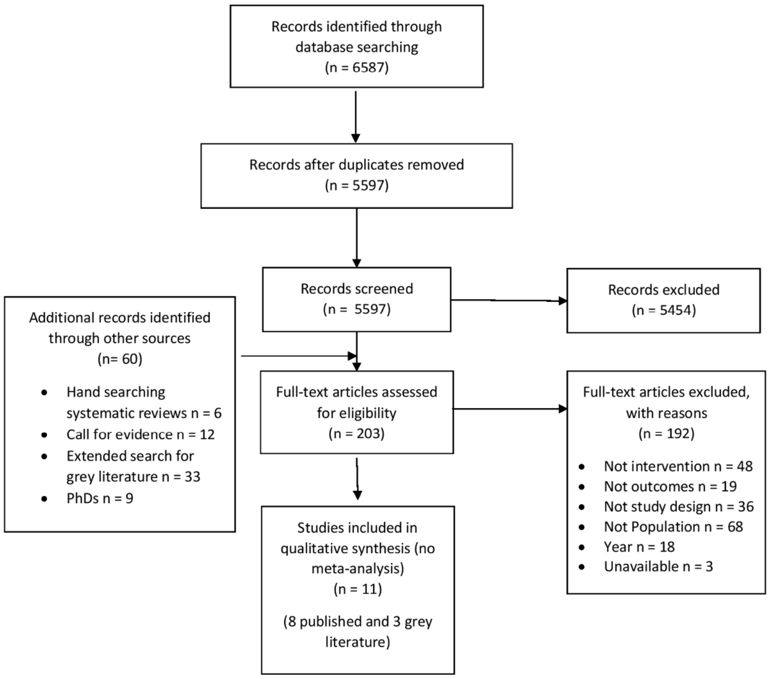

Figure 1 PRISMA flow diagram of the search screening process

210x297mm (200 x 200 DPI)

OVID MEDLINE Search Strategy

1. MeSH descriptor: [well being]

2. well-being

3. wellbeing

4. “young people”.mp or youth.mp or adolescent*.mp

5. sport/ or sport.mp.

6. “physical activity”.mp or “physical activity”/

7. Exercise*.mp.

8. “physical exertion”.mp.

9. dance*.mp.

10. game*.mp.

11. team.mp.

12. bike.mp.

13. cycl*.mp.

14. cheerlead*.mp.

15. equestrian.mp.

16. swim*.mp.

17. gym* .mp.

18. sail*.mp.

19. canoe*.mp.

20. kayak*

21. bloodsport*.mp.

22. boxing.mp

23. “martial arts”.mp.

24. fitness.mp.

25. ballet.mp.

26. choreograph*

27. “work-out”.mp.

28. (1 or 2 or 3) and (4) and (or 5 or 6 or 7 or 8 or 9 or 10 or 11 or 12 or 13 or 14 or 15 or 16 or 17 or 18, or 19 or 20 or 21 or 22 or 23 or 24 or 25 or 26 or 27)

29. tournament.mp

For peer review only

30. match.mp

31. competition.mp

32. festival.mp

33. battle.mp

34. league.mp

35. team*.mp

36. theatre*.mp

37. event*.mp

38. meet*.mp

39. field*.mp

40. fan.mp

41. play*.mp

42. athlet*.mp

43. attend*.mp

44. spectat*.mp

45. particpat*.mp

46. perform*.mp

47. 28 and (29 or 30 or 31 or 32 or 33 or 34 or 35 or 36 or 37 or 38 or 39 or 40 or 41 or 42 or 43 or 44 or 45 or 46)

48. Quality of life.mp. or “Quality of Life”/ Life

49. Anxiety/ or anxiety.mp.

50. self-esteem.mp.

51. loneliness/ or lonel. mp.

52. life adj satisfaction.mp.

53. happiness.mp.

54. worthwhileness.mp.

55. 47 and (48 or 49 or 50 or 51 or 52 or 53 or)

56. limit 54 to humans and all young people or adolescents

For peer review only

Table of excluded studies

Authors	Year	Reason for exclusion
Adie JW, Duda JL, Ntoumanis N.	2008	Intervention
Ahola R, Pyky R, Jämsä T, Mäntysaari M, Koskimäki H, Ikäheimo TM, Huotari ML, Röning J, Heikkinen HI, Korpelainen R.	2013	Study design
Altintas A, Asci FH, Kin-Isler A, Guven-Karahan B, Kelecek S, Ozkan A, Yilmaz A, Kara FM.	2014	Population
Anamaria Constantinescu.	2013	Outcome
Aphamis G, Giannaki CD, Tsouloupas CN, Ioannou Y, Hadjicharalambous M.	2015	Outcome
Aramendi Jauregui P, Bujan Vidales K, Arburua Goyeneche R.	2014	Intervention
Arts Council England	2006	Year
Bamford, C.	2015	Study design
Barton J, Pretty J.	2010	Study Design
Beresford B, Clarke S.	2009	Year
Berntsson LT, Ringsberg KC.	2014	Intervention
BHF National Centre	2014	Study design
Black Country Consortium Ltd	2014	Outcome
Blazy L, Amstel S	NR	Study design
Booker CL, Skew AJ, Kelly YJ, Sacker A.	2015	Intervention
Booker CL, Skew AJ, Sacker A, Kelly YJ.	2014	Intervention
Boyer EM.	2007	Year
Brand S, Gerber M, Beck J, Hatzinger M, Pühse U, Holsboer-Trachsler E.	2010	Intervention
Brassai L, Piko BF, Steger MF.	2011	Intervention
Broďáni J, Spišiak M, Paška Ľ.	2015	Intervention
Brown DR, Carroll DD, Workman LM, Carlson SA, Brown DW.	2014	Population
Buckinghamshire County Council	NR	Study design
Burgess G, Grogan S, Burwitz L.	2006	Population

For peer review only

Casey MM, Harvey JT, Telford A, Eime RM, Mooney A, Payne WR.	2014	Population
Castillo I, Duda JL, Alvarez MS, Merce J, Balaguer I.	2011	Population
Chatzisarantis NLD, Hagger MS.	2007	Intervention
Chen LH, Kee YH.	2008	Intervention
Chen LH, Kee YH, Chen MY.	2015	Outcome
Crossick G, Kaszynska P.	2016	Intervention
Dance is Public Health	2015	Study design
Daniels E, Leaper C.	2006	Intervention
D'anna C, Rio L, Paloma FG.	2015	Intervention
De Bruin AP, Woertman L, Bakker FC, Oudejans RRD.	2009	Intervention
Department of Health, Physical Activity, Health Improvement and Protection	2011	Year
Department of Culture, Arts and Leisure	2009	Year
Di Luzio SS, Procentese F, Guillet-Descas E.	2014	Not available from the British Library
Doerksen SE, Elavsky S, Rebar AL, Conroy DE.	2014	Intervention
Englefield L, Cunningham D, Mahoney A, Stone T, Torrance H.	2016	Outcome
Eime RM, Harvey JT, Brown WJ, Payne WR.	2010	Study design
Falconer C.	2010	Year
Findlay LC, Bowker A.	2009	Population
Fløtnes IS, Nilsen TIL, Augestad LB.	2011	Intervention
Fujiwara D, Kudrna L, Cornwall T, Laffan K, Dolan P.	2015	Outcome
Fujiwara D, Kudrna L, Dolan P.	2014	Population
Fujiwara D, Kudrna L, Dolan P.	2014	Outcome
Fujiwara D, MacKerron G.	2015	Intervention
Gardner SM, Komesaroff P, Fensham R.	2008	Intervention
Geyer J.	2013	Intervention

For peer review only

Gondoh Y, Sensui H, Kinomura S, Fukuda H, Fujimoto T, Masud M, Nagamatsu T, Tamaki H, Takekura H.	2009	Population
Hagensen KP.	2015	Population
Hidalgo-Rasmussen CA, Ramírez-López G, Martín AH-S.	2013	Intervention
HM Government	2016	Outcome
Holland J.	2012	Year
Ivanović M, Milosavljević S, Ivanović U.	2015	Outcome
Jago R, Sebire SJ, Davies B, Wood L, Banfield K, Edwards MJ, Powell JE, Montgomery AA, Thompson JL, Fox KR.	2015	Population
Jalaludin B, Maxwell M, Saddik B, Lobb E, Byun R, Gutierrez R, Paszek J.	2012	Population
Jančiauskas R.	2012	Population
Jelalian E, Hart CN, Mehlenbeck RS, Lloyd-Richardson EE, Kaplan JD, Flynn-O'Brien KT, Wing RR.	2008	Outcome
Jonsdottir IH, Börjesson M, Ahlborg Jr. G.	2011	Population
Jowett GE.	2014	Outcome
Kaczmarek LD, Drążkowski D.	2014	Intervention
Kallings LV, Leijon M, Hellénius M-L, Ståhle A.	2008	Population
Kantor RM, Grimes GR, Limbers CA.	2015	Population
Karadağ Çaman Ö, Özcebe H.	2011	Intervention
Kardefelt-Winther D.	2014	Intervention
Kavetsos G, Szymanski S.	2010	Study design
Kelly P, Matthews A, Foster C.	2012	Year
Kelly NR, Mazzeo SE, Evans RK, Stern M, Thacker LF, Thornton LM, Laver JH.	2011	Population
Kern ML, Waters LE, Adler A, White MA.	2015	Intervention
Khan Y, Taghdisi MH, Nourijelyani K.	2015	Intervention
Kim J, Suh W, Kim S, Gopalan H.	2012	Intervention
Kipp LE, Weiss MR.	2013	Population
Knab AM, Nieman DC, Sha W, Broman-Fulks JJ, Canu WH.	2012	Population

For peer review only

Knifsend CA.	2015	Population
Komlosi, E	2014	Intervention
Kort-Butler LA, Hagewen KJ.	2011	Intervention
Kowert R, Vogelgesang J, Festl R, Quandt T.	2015	Intervention
Lafrenière MA, Vallerand RJ, Donahue EG, Lavigne GL.	2009	Intervention
Laure P, Binsincer C.	2009	Population
Laure P, Binsincer C.	2009	Population
Laurendeau J.	2014	Intervention
Lazaridou A, Kalogianni C.	2013	Outcome
Le Menestrel S, Perkins DF.	2007	Intervention
Lee AJY, Lin WH.	2007	Outcome
Lee BW, Leeson PRC.	2015	Intervention
Leggett, Diane K.	2010	Intervention
Lestan KA, Eržen I, Golobič M.	2014	Population
Leversen I, Danielsen AG, Birkeland MS, Samdal O.	2012	Study Design
Lieber SB, Redberg RF, Blumenthal RS, Gandhi A, Robb KJ, Mora S.	2012	Population
Liu M, Wu L, Ming Q.	2015	Study Design – Systematic Review
Lopez-Walle J, Balaguer I, Castillo I, Tristan J.	2012	Population
Lorger M, Mrakovic S, Hraski M.	2012	Population
LSE Housing and Communities team	2015	Study design
Lu FJH, Hsu YW.	2013	Intervention
Lupu E, Petrescu A.L.	2012	Study Design
Mack DE, Wilson PM, Gunnell KE, Gilchrist JD, Kowalski KC, Crocker PR.	2012	Study design
Madison G, Paulin J, Aasa U.	2013	Population
Maffulli N, Longo UG, Spiezia F, Denaro V.	2010	Intervention
Magnusson M, Hallmyr Lewis M, Smaga-Blom M, Lissner L, Pickering C.	2014	Study Design

For peer review only

Mäkinen M., Lindberg N., Komulainen E., Puukko-Viertomies L.-R., Aalberg V., Marttunen M.	2015	Population
Mancini, JA; Bowen, GL; O'Neal, CW; Arnold, AL	2015	Intervention
Mansfield L, Kay T, Anokye N, Fox-Rushby J.	2015	Study design
Martin-Albo, J; Nunez, JL; Dominguez, E; Leon, J; Tomas, JM	2012	Population
Maugendre M., Spitz E.	2011	Study Design
McDade-Montez, Elizabeth; Wallander, Jan; Elliott, Marc;Grunbaum,Jo Anne; Tortolero, Susan; Cuccaro, Paula; Schuster, Mark A.	2015	Intervention
McGee, R., Williams, S., Howden-Chapman, P., Martin, J. and Kawachi, I	2006	Study design
McMahon E.M., Corcoran P., O'Regan G., Keeley H., Cannon M., Carli V., Wasserman C., Hadlaczky G., Sarchiapone M., Apter A., Balazs J., Balint M., Bobes J., Brunner R., Cozman D., Haring C., Iosue M., Kaess M., Kahn J.-P., Nemes B., Podlogar T., Poštuvan V., Sáiz P., Sisask M., Tubiana A., Värnik P., Hoven C.W., Wasserman D.	2016	Population
Medeiros M.D., De Castro Filho J.A.	2014	Not available from the British Library
Mental Health Foundation	2013	Study design
Merrill R.M., Aldana S.G., Bowden D.E.	2010	Population
Mihaela, Cristuţă Alina	2012	Population
Mochon, D; Norton, MI; Ariely, D	2008	Population
Mohan S., Smith C.A., Corriveau N.L., Kline-Rogers E., Jackson E.A., Eagle K.A., Goldberg C., Durussel-Weston J.	2012	Intervention
Moljord I., Moksnes U.K., Eriksen L., Espnes G.A.	2011	Study Design
Molina J.J.M., Castillo A.S., De La Serrana H.L.G., Díaz M.Z.	2009	Population
Molina-Garcia J, Castillo I, Queralt A	2011	Study Design
Moutão J., Alves S.M., Monteiro D., Cid L.	2015	Population
Nicholls L., Lewis A.J., Petersen S., Swinburn B., Moodie M., Millar L.	2014	Intervention
Noack P., Kauper T., Benbow A.E.F., Eckstein K.	2013	Study Design
Oliver, S.	2009	Year
Optimity Advsors	2016	Population

For peer review only

Orkibi H., Ronen T., Assoulin N.	2014	Population
Papaioannou A.G., Appleton P.R., Torregrosa M., Jowett G.E., Bosselut G., Gonzalez L., Haug E., Ertesvaag V., Zourbanos N.	2013	Population
Peng W., Crouse J.	2013	Outcome
Pérez Ugidos, Guillermo; Laíño, Fernando, A.; Zelarayán, Julio; Márquez, Sara	2014	Intervention
Phillips G Renton A Moore DG Bottomley C Schmidt E Lais S Yu G Wall M Tobi P Frostick C Clow A Lock K Petticrew M Hayes R	2012	Population
Physical Activity Council	2016	Intervention
Piqueras J.A., Kuhne W., Vera-Villarroel P., Van Straten A., Cuijpers P.	2011	Study Design
Play Wales	2012	Year
Precor	NR	Study design
Proctor C., Tsukayama E., Wood A.M., Maltby J., Eades J.F., Linley P.A.	2011	Intervention
Public Health England	2015	Study design
Pyky, R; Jauho, AM; Ahola, R; Ikaheimo, TM; Koivumaa-Honkanen, H; Manysaari, M; Jamsa, T; Korpelainen, R	2015	Intervention
Reding, Frank N; Grieve, Frederick; Derryberry, W. Pitt; Paquin, Anthony R	2011	Outcome
Riley A., Anderson-Butcher D.	2012	Population
Rössler R., Donath L., Verhagen E., Junge A., Schweizer T., Faude O.	2014	Study Design – Systematic Review
Rotheram-Borus M.J., Swendeman D., Becker K.D.	2014	Population
Ryan., K, Mind	2015	Intervention
Sagar, S.S.	2007	Year
Sagatun, A., Søgaard, A.J., Bjertness, E., Selmer, R. and Heyerdahl, S	2007	Study design
Sage, L; Kavussanu, M	2010	Population
Salehi, A; Harris, N; Sebar, B; Coyne, E	2015	Population
Schlarb A.A., Schwedler V., Feichtinger P.	2012	Study Design
Schmiedeberg C., Schröder J.	2016	Population

For peer review only

Schuch F.B., Pinto S.S., Bagatini N.C., Zaffari P., Alberton C.L., Cadore E.L., Silva R.F., Kruel L.F.M.	2014	Population
Schulz, KH; Meyer, A; Langguth, N	2012	Population
Schwanen, T; Wang, DG	2014	Population
Sekot A.	2013	Population
Sellakumar G.K.	2015	Intervention
Shaffer-Hudkins, Emily	2012	Population
Shiue, I	2016	Population
Sidoti E., Paolini G., Tringali G.	2010	Population
Sigvartsen J., Gabrielsen L.E., Abildsnes E., Stea T.H., Omfjord C.S., Rohde G.	2016	Study Design
Sjögren K., Hansson E.E., Stjernberg L.	2011	Population
Skianis, V.	2013	Intervention
Slough Borough Council	NR	Year
Smyth, W.	NR	Population
Snyder A.R., Martinez J.C., Bay R.C., Parsons J.T., Sauers E.L., McLeod T.C.V.	2010	Study Design
Spandler H Mckeown M Roy A Hurley M	2013	Population
Spengler, Sarah; Woll, Alexander	2013	Population
Sport and Recreation Alliance	2012	Year
Sport and Recreation Alliance	2016	Study Design
Stein C., Fisher L., Berkey C., Colditz G.	2007	Population
Stenseng, Frode; Forest, Jacques; Curran, Thomas	2015	Population
StreetGames	2016	Study design
Stubbe J.H., de Moor M.H.M., Boomsma D.I., de Geus E.J.C.	2007	Population
Student Sport Ireland	2016	Outcome
Suendermann, S.,	2015	Population
Sztankovics A.	2013	Population
Tanimaru J.H., Dos Santos A.L.P.	2016	Study Design

For peer review only

Taylor, P., Davies, L., Wells, P., Gilbertson, J. & Tayleur, W.	2015	Study design
Tharenos C.L., Santorino D.	2009	Not available from the British Library
The Department of Culture, Arts and Leisure	2009	Year
Thøgersen-Ntoumani C., Ntoumanis N.	2006	Population
Thomley B.S., Ray S.H., Cha S.S., Bauer B.A.	2011	Population
TOP Foundation	2014	Study design
UK Community Foundations	2012	Year
Urmston, E.	2012	Year
Urmston, E.	2013	Population
Vilela C., Gomes A.R.	2015	Intervention
Wall M., Hayes R., Moore D., Petticrew M., Clow A., Schmidt E., Draper A., Lock K., Lynch R., Renton A.	2009	Study design
Watson, B., Lashua, B., Trevorrow, P.	2016	Outcome
Whitehead, S.H.	2005	Year
Wicker, P; Coates, D; Breuer, C	2015	Population
Wicker, P; Frick, B	2015	Population
Williams K., Davis III O., Gittelman M., Pomerantz W.J.	2006	Population
Williams, G. & Jacques, K.	NR	Population
Woodall, J; White, J; South, J	2013	Population
Yamada K., Kawata Y., Nakajima N., Hirosawa M.	2012	Outcome
Zook K.R., Saksvig B.I., Wu T.T., Young D.R.	2014	Outcome
Zullig, Keith J.; White, Rebecca J.	2011	Population

For peer review only

Published Literature Data Extraction Form

Reviewer Initials:

**Title, Author, year**	
**Study objectives**	
**Study design**	
**Method of allocation to study group**	
**Outcomes and measures used (relevant to review)** (Include scale(s) used and time-points)	
**Intervention** (brief description of the intervention used)	
**Details of analysis** (Include type of analysis i.e. quantitative/qualitative/mixed, and method and/or process of analysis e.g. thematic analysis/statistical analysis, any subgroup analysis and any methods used in the treatment of missing data)	
**Participants included (at baseline and follow up in each group)** (Source/recruitment, eligible and selected, number, age restrictions, exclusions, gender)	
**Intervention(s) and comparison group(s)** (Type, content, intervener, duration, method, mode or timing of delivery)	
**Results** (Key numerical results including proportions experiencing relevant outcomes in each group, means, medians, standard deviations, ranges and effect sizes with precision estimates e.g. confidence intervals/ p values whether or not significant [if P values are not reported this should be stated]. For qualitative data what categories/themes were found, results drawn by authors and evidence provided. Identify any inadequately reported missing data	
**Protected characteristics** (Methods and findings that relate to protected characteristics [age, sex, gender reassignment, sexual orientation, disability, race, religion, pregnancy/maternity, marriage/civil partnerships] and income and/or socio-economic status.	
**Limitations identified**	

For peer review only

**Review conclusions** (for each comparison made)	
**Conflicts of interest and sources of funding**	
**Ethical procedures reported**	
**Grade/CERQual Rating**	

GRADE and CERQual for judging certainty / quality of evidence

Quantitative: Grade

**Type of evidence**	Randomized trial = high<br>Observational study = low<br>Any other evidence = very low
**Decrease grade if (Each quality criteria can reduce the quality by one or, if very serious, by two levels.)**	• Serious or very serious limitation to study quality (e.g. Important inconsistency; major uncertainty about directness; imprecise or sparse data; high probability of reporting bias
**Increase grade if**	• Strong evidence of association—significant relative risk of > 2 (< 0.5) based on consistent evidence from two or more observational studies, with no plausible confounders (+1)<br>• Very strong evidence of association—significant relative risk of > 5 (< 0.2) based on direct evidence with no major threats to validity (+2)<br>• Evidence of a dose response gradient (+1)<br>• All plausible confounders would have reduced the effect (+1)
**Grade Rating / Range**	High quality evidence<br>Moderate quality evidence<br>Low quality evidence<br>Very low quality evidence

Qualitative: CERQual

**Increase confidence if**	• Study is well designed with few limitations<br>• Evidence applicable to context (perspective or population, phenomenon of interest, setting) specified in objectives<br>• Findings/conclusions supported by evidence and provide convincing explanation for patterns found<br>• Data supporting findings is rich and good quality
**Decrease confidence if** (Each quality criteria can reduce the quality by one or, if very serious, by two levels)	• Serious or very serious limitations in design or conduct of the study<br>• Evidence is not relevant to the study objectives<br>• Findings/conclusions are not supported by the evidence<br>• Data is poor quality and inadequate to support findings
**CERqual Confidence Rating / Range**	**High confidence** It is highly likely that the review finding is a reasonable representation of the phenomenon of interest<br>**Moderate confidence** It is likely that the review finding is a reasonable representation of the phenomenon of interest<br>**Low confidence** It is possible that the review finding is a reasonable representation of the phenomenon of interest<br>**Very low confidence** It is not clear whether the review finding is a reasonable representation of the phenomenon of interest.

For peer review only

Grey literature data extraction tool

Part 1. Project details

Author details
Record of the authors' details, date of publication, title of the report, publisher and place of publication.
Project aims
Include aims and objectives for the project.
Project partners
Record the organisations involved in project delivery. Who is the lead delivery partner who managed the intervention?
Commissioner(s) and funding sources
Who funded the project?
Type of arts or sport intervention
E.g. music, singing, dance etc.
Project description
For how long did the intervention run? How many sessions, episodes or events were delivered? What were the delivery dates? Record the key components, activities and events. Include details of equipment needed to run the intervention and staff competencies of those delivering it. Where did the project take place? Include geographical location and type of setting, e.g. museum, college, sports centre. It is important to record any special conditions, such as incentives or access to prestigious venues that may have affected participants' experiences of the project.
Target population
Who was the target population? Include age, gender, ethnicity, demographic details, health conditions and localities if relevant. How were participants recruited to the intervention? E.g. referral process or is it self-selecting? How many people actually took part?
Project costs
Record details of project costs, including costs per participant, and costs to participants, if reported.

For peer review only

Part 2: Evaluation details

Evaluation aims and objectives
What was the rationale for the evaluation? What key outcomes and impacts were prioritised for evaluation. What questions did the evaluation seek to address? Did it build on previous work, e.g. a theory of change/logic model/evidence review/research study or previous evaluation?
Conducting the evaluation
Who conducted the evaluation? Who managed it and was responsible for any changes in the design or responding to adverse events?
Type of evaluation and evaluation design
E.g. basic monitoring, process evaluation, quantitative, qualitative, mixed methods etc.
Evaluation budget
What resources were set aside for evaluation? What was the duration of evaluation funding, if this was received?
Data collection procedures
Provide details of quantitative and qualitative data collection procedures.
Sampling, selection and recruitment of participants
How were participants selected for data collection, including qualitative interviews, focus groups and case studies? How many actually people took part?
Evaluation timeline
When were the data collected?
Ethics and consent
What were the ethical considerations for the evaluation? Was the anonymity of participants be protected? Were the participants particularly vulnerable? Was formal ethics approval obtained?
Data analysis
How were the data analysed? Were there any biases in data analysis and reporting?
Key findings
What wellbeing outcomes were reported? How was wellbeing reflected in qualitative themes?
Findings from process evaluation
What broader impacts or learning were recorded?

Reference

Adapted from Public Health England Arts and Health Evaluation Framework
https://www.gov.uk/government/uploads/system/uploads/attachment_data/file/496230/PHE_Arts_and_Health_Evaluation_FINAL.pdf

For peer review only

Quality checklist: Quantitative evidence of intervention effectiveness

How to use this checklist: This checklist is to be used to indicate if a specific study has been well designed, appropriately carried out and analysed, i.e. the confidence we can have in the results of whether an intervention was effective. This should be used for the summary table, to make brief comments on the risk of bias of each study. In turn, the overview of the <u>study</u> limitations will help to inform the quality of the overall <u>body</u> of evidence.

**Evidence quality of intervention effectiveness / study limitations**				
**1. Was the evaluation <u>well-designed</u>?**	**Yes**	**No**	**Can't tell**	**N/A**
• **Fidelity:** The extent to which the intervention was delivered with fidelity is clear - i.e. if there is a specific intervention which is being evaluated, this has been well reproduced.<br>• **Measurement**: The measures are appropriate for the intervention's anticipated outcomes and population.<br>• Participants completed the same set of measures once shortly before participating in the intervention and once again immediately afterwards<br>• An 'intent-to-treat' design was used, meaning that all participants recruited to the intervention participated in the pre/post measurement, regardless of whether or how much of the intervention they received, even if they dropped out of the intervention (this does not include dropping out of the study- which may then be regarded as missing data)<br>• **Counterfactual**:<br>• Assignment to the treatment and comparison group was at the appropriate level (e.g., individual, family, school, community)<br>• The comparison condition provides an appropriate counterfactual to the treatment group. Consider**:**<br>o Participants were randomly assigned to the treatment and control group through the use of methods appropriate for the circumstances and target population OR sufficiently rigorous quasi-experimental methods (regression discontinuity, propensity score matching) were used to generate an appropriately comparable sample through non-random methods<br>o The treatment and comparison conditions are thoroughly described.				

2. Was the study carried out appropriately? including appropriate sample	Yes	No	Can't tell	N/A
• **Representative:** The sample is representative of the intervention's target population in terms of age, demographics and level of need. The sample characteristics are clearly stated.<br>• There is baseline equivalence between the treatment and comparison group participants on key demographic variables of interest to the study and baseline measures of outcomes (when feasible)<br>• **Sample size**: The sample is sufficiently large to test for the desired impact. This depends most importantly on the effect size, however a suggestion could be e.g. a minimum of 20 participants have completed the measures at both time points within each study group.<br>• **Attrition:** A minimum of 35% of the participants completed pre/ post measures. Overall study attrition is not higher than 65%.<br>• The study had clear processes for determining and reporting drop-out and dose. Differences between study drop-outs and completers were reported if attrition was greater than 10%.<br>• The study assessed and reported on overall and differential attrition<br>• **Equivalence:** Risks for contamination of the comparison group and other confounding factors have been taken into account and controlled for in the analysis if possible:<br>o Participants were blind to their assignment to the treatment and comparison group<br>• There was consistent and equivalent measurement of the treatment and control groups at all points when measurement took place.<br>• **Measures**: The measures used were valid and reliable. This means that the measure was standardised and validated independently of the study and the methods for standardization were published. Administrative data and observational measures may also have been used to measure programme impact, but sufficient information was given to determine their validity for doing this.<br>• Measurement was independent of any measures used as part of the treatment.<br>• In addition to any self-reported data (collected through the use of validated instruments), the study also included assessment information independent of the study participants (eg, an independent observer, administrative data, etc).				

3. Was analysis appropriate?	Yes	No	Can't tell	N/A
• The methods used to analyse results are appropriate given the data being analysed (categorical, ordinal, ratio/ parametric or non-parametric, etc) and the purpose of the analysis.<br>• Appropriate methods have been used and reported for the treatment of missing data.				
**4. Is the evidence consistent?**				
• Are the findings made explicit?<br>• Is there adequate discussion of the evidence both for and against the researcher's arguments?<br>• Has the researcher discussed the credibility of their findings (e.g. triangulation, respondent validation, more than one analyst)?<br>• Are the findings discussed in relation to the original research question?				

Table of Subjective Wellbeing Measures used in Included Studies

Measurement tool	Outcome measuring	Description	Scoring/ interpretation	Validity & Reliability
Rosenberg's Self-Esteem Scale	Self-esteem	10-item scale that measures global self-worth by measuring both positive and negative feelings about the self. The scale is believed to be uni-dimensional. All items are answered using a 4-point Likert scale format ranging from strongly agree to strongly disagree. Five of the items have positively worded statements and five have negatively worded ones. The scale measures state self-esteem by asking the respondents to reflect on their current feelings.	Range: 0-30<br>15- 25 normal range;<br>below 15 low self-esteem.	The original sample for which the scale was developed in the 1960s consisted of 5,024 high school juniors and seniors from 10 randomly selected schools in New York State and was scored as a Guttman scale. The scale generally has high reliability: test-retest correlations are typically in the range of .82 to .88, and Cronbach's alpha for various samples are in the range of .77 to .88.
Athlete Burnout Questionnaire (Raedeke & Smith 2001)	Athletes level of Burn out	15 item assessing 3 dimensions of burnout:<br>-Emotional/physical exhaustion<br>-Reduced sense of accomplishment<br>-Sport devaluation<br><br>The stem for each item is “How often do you feel this way?” Each response is scored on a 5-point Likert scale: “almost never” (1), “rarely” (2), “sometimes” (3), “frequently” (4), “almost always” (5).	Combined scores from each item for a dingle global indicator<br>(higher the score the higher the level of burnout)	Raedeke and Smith (2001) and Cresswell and Eklund (2006) demonstrated reliability and validity both in and out of North America
Beck Depression Inventory first published in 1961, revised in 1978 (BDI-1A) and then 1996 (BDI-II)	Depression (presence and degree. NOT a diagnostic instrument)	21-question multiple-choice self-report inventory for adolescents and adults. Evaluates 21 symptoms of depression (15 on emotions, 4 on behavioural changes, 6 on somatic symptoms).<br>The 21 items cover sadness, pessimism, past failure, self-dislike, self-criticism, suicidal thoughts or wishes, crying,	0–9 not depressed<br>10–18 mild-moderate depression<br>19–29 moderate-severe<br>30–63 severe<br><br>According to paper:<br>0–9 normal<br>10–15 low	Beck reviewed 11 studies and the BDI was capable of discriminating between groups that contrasted in level of depression. Beck’s original paper reported an internal consistency studies demonstrated a correlation coefficient of .86 for the test items, and the Spearman-

For peer review only

		agitation, loss of interest, indecisiveness, worthlessness, loss of energy, changes in sleeping patterns, irritability, changes in appetite, difficulty concentrating, tiredness or fatigue, and loss of interest in sex.<br>Time to Administer: 5-10 minutes	16–23 medium<br>24+ depressive	Brown correlation for the reliability of the BDI yielded a coefficient of .93.<br>Criticisms; BDI-IA only addresses six out of the nine DSM-III criteria for depression, self-reported (reporting bias), questionnaire therefore the way administered could affect outcome e.g. social desirability. If pt has a physical illness the physical symptoms such as fatigue may score higher but not reflect depression.
Subjective Exercise Experiences Scale (SEES)	Measuring 3 dimensions; positive well-being, psychological distress, and fatigue	"By circling a number on the scale below each of the following items, please indicate the degree to which you are experiencing each feeling now, at this point in time, after exercising". Each item rated on a 7-point Likert scale: 1 (Not at all) - 7 (Very much so).<br>12 item scale (4 items per dimension): great, awful, drained, positive, crummy, exhausted, strong, discouraged, fatigued, terrific, miserable, and tired.	The Items (4 items per dimension) are summed to create a summary score for Positive Well-Being, Psychological Distress and Fatigue. Therefore each dimension has a possible score up to 28, the higher the number the higher the association with the trait.	Validity and reliability have been reported for other groups (McAuley & Courneya, 1994; Rudolph & Kim, 1996).
Positive Affect Negative Affect Schedule (PANAS) (Watson, Clark, & Tellegen, 1988)	Hedonic Well-Being/ the intensity associated with both positive and negative dimensions of global affect	20-item self-report instrument. Rate each using a 5-point Likert scale ranging from 1 (Not At All or Very Slightly) to 5 (Very Much).<br><br>PANAS for Children (PANAS-C): 30-item measure (15 positive affect and 15 negative affect items). Indicate how often they have felt interested, sad, and so on during the "past few weeks" on a 5-point Likert scale ranging from 1 (very slightly or not at all) to 5 (extremely).	Positive Affect Score:<br>range from 10 – 50, with higher scores representing higher levels of positive affect.<br>Negative Affect Score:<br>ranges from 10 – 50, with lower scores representing lower levels of negative affect.<br><br>PANAS-C:<br>Summation scores for positive affect and negative affect range from 10 to75 each.	Reliability and Validity reported by Watson (1988) was moderately good. For the Positive Affect Scale, the Cronbach alpha coefficient was 0.86 to 0.90; for the Negative Affect Scale, 0.84 to 0.87. Over a 8-week time period, the test-retest correlations were 0.47-0.68 for the PA and 0.39-0.71 for the NA. The PANAS has strong reported validity with such measures as general

For peer review only

				distress and dysfunction, depression, and state anxiety.<br><br>PANAS-C has demonstrated good convergent and discriminant validity in adolescent samples
Self-Esteem Scale (SES)	Self-esteem	10 items, and the total score ranges from 10 to 40.	Higher scores = higher self-esteem	NR
Profile of Mood States (POMS) scale	Mood & mindfulness	7 subscales (tension, anger, fatigue, depression, vigor, confusion, and mood related to self-esteem) with 40 adjectives that describe mood.<br><br>Original: 65 adjectives rated on 5-point scale<br>0= not at all; 1=a little; 2=moderately; 3=quite a bit; 4=extremely<br><br>Short Form (POMS-SF): 30-item consisting of 30 adjectives rated on a 5-point scale ranging from 0 (not at all) to 4(extremely).	Higher scores (POMS Total Mood Disturbance (TMD)) = more negative current mood states<br><br>POMS-SF:<br>Responses are summed (with positive items reverse scored) to provide a TMD score (range 0–100), as well as subscale scores for 6 mood states (each ranging 0–20): Tension-Anxiety, Depression-Dejection, Anger-Hostility, Vigor-Activity, Fatigue-Inertia, and Confusion-Bewilderment.	High internal consistency of subscales and validity for original POMS scale
WHOQOL-BREF Scale	QoL	The World Health Organization Quality of Life (WHOQOL). WHOQOL-BREF is a shorter version containing 26 items (1 from each of the 24 facets in the WHOQOL-100 plus 2 items from the Overall quality of Life and General Health facet) measuring these domains: physical health, psychological health, social relationships, and environment.	QoL profile with 4 domain scores plus overall perception of QoL and overall perception of health. Higher scores = higher QoL.	developed by the WHOQOL Group with<br>fifteen international field centres, simultaneously, in an attempt to develop a quality of life assessment that<br>would be applicable cross-culturally
Schulte Grid	Attention	A Schulte table (8*8 grid) is a square that consists of 64 squares of the same size (1 × 1 cm), with one of 64 random numbers from 1 to 64. When tested, individuals are required to figure out the numbers in the	Less time represents higher level of attention	NR

For peer review only

		order from 1 to 64, and read out the numbers loud at the same time. Timing starts with 1 and ends with 64.		
Perceived stress scale (PSS) (Cohen et al, 1983).<br><br>Chinese Perceived Stress Scale (CPSS)	Stress	10-items measuring the degree to which events are appraised as stressful during the past month. Items rated on a Likert scale from 0 (never) to 4 (very often).<br>Items designed to tap how unpredictable, uncontrollable, and over-loaded respondents find their lives. The scale also includes several direct queries about current levels of experienced stress.<br><br>CPSS-14 questions	Responses summed to give a total score ranging from 0 to 40 (CPSS: 0-56).<br><br>Higher composite scores indicate greater perceived stress.	The PSS is the most widely used psychological instrument for measuring the perception of stress.<br>CPSS-10 showed a stable two-factor structure with satisfactory internal consistency and construct validity (Siu-man Ng, 2013)
Inventory of Positive Psychological Attitudes	Positive worldview, Confidence in Life and Self (two sub-scales: Life Purpose and Satisfaction (LPS) and Self-Confidence During Stress (SCDS)).	32-item, 7-point Likert self-report scale. Example questions: Life Purpose and Satisfaction Section: My daily activities are -<br>Response: not a source of satisfaction to a source of satisfaction (7 pt scale)<br>Self-Confidence During Stress Section: When there is a great deal of pressure being placed on me - Response: I get tense to I remain calm (7 pt scale).	Each score is calculated as a mean; possible scores ranging from 1 to 7. VERY LOW: 1.00 TO 2.49<br>MEDIUM LOW: 2.50 TO 3.99<br>MEDIUM HIGH: 4.00 TO 5.49<br>VERY HIGH: 5.50 TO 7.00	It has been shown to possess adequate reliability and construct validity in samples of undergraduate college students.
Resilience Scale	self-regulatory skills (degree of individual resilience)	25-item covering 5 factors of resilience; meaningful life (purpose); perseverance; self-reliance; equanimity; and coming home to yourself (existential aloneness). Items scored on a 7-point scale from 1 (disagree) to 7 (agree)	Possible scores ranging from 25 to 175. Higher scores reflect greater resilience.	The scale has internal consistency, reliability, and concurrent validity and has been recommended as the best instrument for measuring resilience in adolescents (Ahern et al, 2006).
Child Acceptance Mindfulness Measure	self-regulatory skills (mindfulness)	25-item measure assessing the degree to which children and adolescents observe internal experiences, act with awareness,	A total score is calculated by reverse scoring negatively worded items and summing the item total. Range in scores from 0 to 100.	The CAMM has demonstrated good internal consistency and concurrent validity with negative correlations to measures of

For peer review only

		and accept internal experiences without judging them.	Higher scores indicate higher levels of acceptance and mindfulness.	cognitive suppression and psychological inflexibility in a study of 606 middle school students (Coyne, Cheron & Ehrenreich, 2008)
State Trait Anger Expression Inventory-2TM (Spielberger, 1999)	self-regulatory skills (experience, expression, and control of anger)	Designed for people aged 16 years and older.<br>57-item self-report tool with a 4-point Likert response format. The instrument is categorized into subscales that reflect state anger (3 subscales), trait anger (2 subscales), and anger expression ().<br><br>Study reported in used sub scales measuring anger expression. Anger expression was conceptualized as having 3 major components: anger-out (outward expression of anger), anger-in (anger suppression), and anger control (attempts to control expression of anger)	For each scale, summation scores range from 8 to 32.<br><br>Higher the score = stronger association. Higher Anger-in = more negative anger expression, higher anger-out = more negative anger expression, higher anger control = better anger control.	Strong reliability and validity. STAXI-2 has been shown to be a suitable instrument to measure both the experience and the expression of anger in both general and clinical populations (Lievaart, Franken, Hovens, 2014).
Friendship Quality Questionnaire	Peer support	assess the quality of children's and early adolescents' relationships with their best friends according to five dimensions: companionship, conflict, help/aid, security and closeness.	NR	A confirmatory factor analysis, used to evaluate the factor structure of this instrument, demonstrated that these scales represented distinct, but related, domains of friendship. Assessments of reliability indicated the high level of internal consistency within each dimension. The validity of the scale was indicated by the observation of higher ratings for (a) mutual friends than for non-mutual friends, and (b) for stable friends than for non-stable friends.

For peer review only - http://bmjopen.bmj.com/site/about/guidelines.xhtml

Exercise Confidence Survey	Self-efficacy	8 items each on a 10 pt likert scale (I know I can, to 10 I know I cannot)	Total the numbers circled and the higher the score, the less likely you are to stick with your exercise program.	
-5 item Subscale of the Intrinsic Motivation Inventory	Need Satisfaction (sport competence)	5 items – rated on 7 pt Likert scale (1 strongly disagree – 7 strongly agree)	Higher score = agree more	Each has shown adequate psychometric properties with adolescent athletes in similar studies testing SDT (Amarose & Anderson-Butcher 2007)
-6 item Scale (developed by Hollembeak & Amorose, 2005)	Need Satisfaction (need for autonomy)	the measure asks respondents to indicate the amount of choice or control they have when participating in their current sport. 6 items - rated on a range from 1 (not at all true) to 5 (completely true)		
-Sport oriented version of Richer & Vallerand's Feelings of Relatedeness Scale	Need Satisfaction (need for relatedeness)	rate the extent they agree with a series of 10 adjectives describing their relationships with members of their sport team. Range from 1 (do not agree at all) to 7 (very strongly agree)		
-Anger (16 item questionnaire)	Anger	questionnaires were developed by DIPAS (Defense Institute of Physiology and Allied Sciences), New Delhi, India. Every item amongst all the questionnaires measures the tested domain on the weighted scores of responses from 0 (never) to 3 (almost always).	Questionnaires were scored by adding the weighted (0 to 3) scores of each item.<br><br>Sense of well-being: The lesser the score the better is the sense of well-being	These questionnaires were chosen as they are valid for Indian population, reliable and specific to measure the tested psychological domains.
-Trait anxiety (40 item questionnaire)	Anxiety			
-Depression (10 item questionnaire)	Depression (incl. depressed mood, guilt, difficulty in sleeping, decision making, work and interests)			
-Subjective well-being (50 item questionnaire)	Subjective WB (incl. the ability to			

For peer review only

	develop persons' potential; work productivity and creativity; build strong and positive relationships with others)			
The Swedish version of a 10-item General Self-Efficacy Scale (GSES)	Self-efficacy	The Swedish version of the 10-item General Self-Efficacy Scale (GSES), developed by Koskinen-Hagman, Schwartzer and Jerusalem. Original version used a 4-point Likert scale, but a pilot test demonstrated that was too limited to detect variations in participants' responses. This scale was extended to a 6-point Likert scale	The total score is calculated by finding the sum of all items. The total score ranges between 10and 60, with a higher score indicating more self-efficacy. This paper argues that lower score indicates a higher perceived GSE. (note original scale says higher score = higher S-E)	GSES is correlated to emotion, optimism, work satisfaction. Negative coefficients for depression, stress, health complaints, burnout, and anxiety. Internal reliability for GSE = Cronbach's alphas between .76 and .90
Swedish version - Social Barriers to Exercise Self-Efficacy Questionnaire (SPBESQ)	Behaviour specific self-efficacy	6-point Likert scale. Response ranges from 1 'not true' to 6 'absolutely true'.10 items (3 for support barriers and 7 for social barriers). Examines content of intervention and specific behavioural changes.	A lower score appears to suggest a higher perceived SSBES in this paper (note lack of clarity re: direction of effect)	NR

For peer review only

PRISMA 2009 Checklist

Section/topic	#	Checklist item	Reported on page #
**TITLE**			
Title	1	Identify the report as a systematic review, meta-analysis, or both.	1, 2
**ABSTRACT**			
Structured summary	2	Provide a structured summary including, as applicable: background; objectives; data sources; study eligibility criteria, participants, and interventions; study appraisal and synthesis methods; results; limitations; conclusions and implications of key findings; systematic review registration number.	2
**INTRODUCTION**			
Rationale	3	Describe the rationale for the review in the context of what is already known.	3-4
Objectives	4	Provide an explicit statement of questions being addressed with reference to participants, interventions, comparisons, outcomes, and study design (PICOS).	8
**METHODS**			
Protocol and registration	5	Indicate if a review protocol exists, if and where it can be accessed (e.g., Web address), and, if available, provide registration information including registration number.	2
Eligibility criteria	6	Specify study characteristics (e.g., PICOS, length of follow-up) and report characteristics (e.g., years considered, language, publication status) used as criteria for eligibility, giving rationale.	5, 8
Information sources	7	Describe all information sources (e.g., databases with dates of coverage, contact with study authors to identify additional studies) in the search and date last searched.	6
Search	8	Present full electronic search strategy for at least one database, including any limits used, such that it could be repeated.	Appendix 1
Study selection	9	State the process for selecting studies (i.e., screening, eligibility, included in systematic review, and, if applicable, included in the meta-analysis).	6-7
Data collection process	10	Describe method of data extraction from reports (e.g., piloted forms, independently, in duplicate) and any processes for obtaining and confirming data from investigators.	9
Data items	11	List and define all variables for which data were sought (e.g., PICOS, funding sources) and any assumptions and simplifications made.	8
Risk of bias in individual studies	12	Describe methods used for assessing risk of bias of individual studies (including specification of whether this was done at the study or outcome level), and how this information is to be used in any data synthesis.	9-11
Summary measures	13	State the principal summary measures (e.g., risk ratio, difference in means).	11
Synthesis of results	14	Describe the methods of handling data and combining results of studies, if done, including measures of consistency (e.g., I^2) for each meta-analysis.	11

For peer review only - http://bmjopen.bmj.com/site/about/guidelines.xhtml

PRISMA 2009 Checklist

Section/topic	#	Checklist item	Reported on page #
Risk of bias across studies	15	Specify any assessment of risk of bias that may affect the cumulative evidence (e.g., publication bias, selective reporting within studies).	n/a
Additional analyses	16	Describe methods of additional analyses (e.g., sensitivity or subgroup analyses, meta-regression), if done, indicating which were pre-specified.	n/a
**RESULTS**			
Study selection	17	Give numbers of studies screened, assessed for eligibility, and included in the review, with reasons for exclusions at each stage, ideally with a flow diagram.	6
Study characteristics	18	For each study, present characteristics for which data were extracted (e.g., study size, PICOS, follow-up period) and provide the citations.	12-23
Risk of bias within studies	19	Present data on risk of bias of each study and, if available, any outcome level assessment (see item 12).	9 and table 2 & 3
Results of individual studies	20	For all outcomes considered (benefits or harms), present, for each study: (a) simple summary data for each intervention group (b) effect estimates and confidence intervals, ideally with a forest plot.	Table 3 pp 12-16 and pp 26-28
Synthesis of results	21	Present results of each meta-analysis done, including confidence intervals and measures of consistency.	No meta analysis – narrative presentation pp. 26-28
Risk of bias across studies	22	Present results of any assessment of risk of bias across studies (see Item 15).	n/a
Additional analysis	23	Give results of additional analyses, if done (e.g., sensitivity or subgroup analyses, meta-regression [see Item 16]).	n/a
**DISCUSSION**			
Summary of evidence	24	Summarize the main findings including the strength of evidence for each main outcome; consider their relevance to key groups (e.g., healthcare providers, users, and policy makers).	28
Limitations	25	Discuss limitations at study and outcome level (e.g., risk of bias), and at review-level (e.g., incomplete retrieval of identified research, reporting bias).	29
Conclusions	26	Provide a general interpretation of the results in the context of other evidence, and implications for future research.	30
**FUNDING**			

For peer review only

PRISMA 2009 Checklist

Funding	27	Describe sources of funding for the systematic review and other support (e.g., supply of data); role of funders for the systematic review.	31

From: Moher D, Liberati A, Tetzlaff J, Altman DG, The PRISMA Group (2009). Preferred Reporting Items for Systematic Reviews and Meta-Analyses: The PRISMA Statement. PLoS Med 6(7): e1000097. doi:10.1371/journal.pmed1000097

For more information, visit: **www.prisma-statement.org**.

For peer review only

For peer review only - http://bmjopen.bmj.com/site/about/guidelines.xhtml

PEER REVIEW HISTORY

BMJ Open publishes all reviews undertaken for accepted manuscripts. Reviewers are asked to complete a checklist review form (**http://bmjopen.bmj.com/site/about/resources/checklist.pdf**) and are provided with free text boxes to elaborate on their assessment. These free text comments are reproduced below.

ARTICLE DETAILS

**TITLE (PROVISIONAL)**	Sport and dance interventions for healthy young people (15-24 years) to promote subjective wellbeing: A systematic review
**AUTHORS**	Mansfield, Louise; Kay, Tess; Meads, Catherine; Grigsby-Duffy, Lily; Lane, Jack; John, Alistair; Daykin, Norma; Dolan, Paul; Testoni, Stefano; Julier, Guy; Payne, Annette; Tomlinson, Alan; Victor, Christina

VERSION 1 – REVIEW

**REVIEWER**	patrizia calella<br>parthenope university, naples
**REVIEW RETURNED**	06-Jan-2018

**GENERAL COMMENTS**	The authors aimed to describe the subjective wellbeing with sport and dance interventions in healthy young people.<br>This is important and relevant in order to understant the sport and dance impact on wellbeing. However, the review is too redundant on some section like the metodology and there is not a clear discussion section, which made it difficult to contexualize the review in the scientific literature.<br>Therefore, in order to improve the paper, some minor review is suggested:<br>1) the box at the beginning of the articles is redundant with the last paragraphs of the discussion<br>2) revise the order of the tables in the manuscript to be sure that they are immediately after the section they are cited<br>3) revise all the table to be sure that the information are in the same order and in the same format<br>4) explain the acronyms presented in the tables<br>5) the discussion section need to be improved with some comparison with other studies in the scientific literature<br>See all details in the attached file<br><br>- The reviewer also provided a marked copy with additional comments. Please contact the publisher for full details.

**REVIEWER**	Brenda Happell<br>University of Canberra, Australia
**REVIEW RETURNED**	28-Jan-2018

**GENERAL COMMENTS**	Thanks for the opportunity to review this paper. It is well written and deals with an important topic. Ways to positively influence the well-being of young people is crucial in promoting optimal mental and physical health. I was very pleased to see gray literature included.

	The introduction could be strengthened with a stronger rationale for the review.<br>p. 3 is 'worthwhileness' a word?<br><br>Methods:<br>Suggest a justification is provided for the timespan of the review. Otherwise very comprehensive and rigourous section,<br><br>Results:<br>Well presented, easy to follow<br><br>Discussion:<br>This section needs the most work. As written it is more like a summary of the results. These need to be clearly related to the broader literature. What does this all mean? How can this knowledge be utilised? How does it relate to what we already know?<br><br>I encourage the authors to make these changes and good luck with your future work.

**REVIEWER**	Greg Atkinson<br>Health and Social Care Institute, Teesside University, UK
**REVIEW RETURNED**	07-Feb-2018

**GENERAL COMMENTS**	I was asked to review this systematic review from the perspective of statistical analysis. However, the authors have stated that the interventions and outcome measures were too variable to undertake a robust meta-analysis. Therefore, there are no real statistical issues to scrutinise in my opinion. I have read the various study descriptions and I do agree that the outcomes in particular are heterogeneous and therefore I do agree that I do not think a meta-analysis is warranted in this particular case.

**REVIEWER**	Ale McConnachie<br>Robertson Centre for Biostatistics<br>University of Glasgow<br>Scotland
**REVIEW RETURNED**	20-Feb-2018

**GENERAL COMMENTS**	Mansfield et al report a systematic review of sport and dance intervention to improve wellbeing in health young people. This review considers the statistical aspects of the paper.<br><br>The paper is well written and tells a coherent story. The authors decide that due to the variabiity between the studies reported, a meta analysis would not be appropriate. This is fully acceptable. That being the case, there is very little for me to comment on in the paper. As far as I can tell, this is a good example of a narrative systematic review, but this is not my area of expertise.

VERSION 1 – AUTHOR RESPONSE

Response to Reviews

Reviewers 3 and 4

Reviewers 3 and 4 provided expert statistical review on the paper and we thank them for their comments. Both agree that our decision not to conduct a meta-analysis due to the variability in the interventions and outcome measures in the studies is fully acceptable.

Reviewer 1

The authors aimed to describe the subjective wellbeing with sport and dance interventions in healthy young people. This is important and relevant in order to understand the sport and dance impact on wellbeing. However, the review is too redundant on some section like the methodology and there is not a clear discussion section, which made it difficult to contexualize the review in the scientific literature.

Therefore, in order to improve the paper, some minor review is suggested:
1) the box at the beginning of the articles is redundant with the last paragraphs of the discussion

Thank you for noting the repetition. We have deleted the text at the end of the article and ensured all information is in the box at the beginning as it is our understanding the box is a requirement for BMJ publications

2) revise the order of the tables in the manuscript to be sure that they are immediately after the section they are cited

Thank you for noting the inconsistency. We have reviewed the position of all tables and moved table 1 to the appropriate place after the section in which it is cited. Table 1 now appears on page 7.

3) revise all the table to be sure that the information are in the same order and in the same format

We agree that consistency in formatting of the table is essential and have reviewed and edited accordingly. It is table 3 (characteristics of included studies) and table 4 (summary of numerical results of included studies) that have been specifically edited to respond to this point. In the revised manuscript we include the corrected tables with no track changes. We have uploaded tables 3 and 4 with track changes showing as separate documents for reviewers to see the edits.

4) explain the acronyms presented in the tables

A clearer key to acronyms is not included in the edited tables

5) the discussion section need to be improved with some comparison with other studies in the scientific literature

We agree that the discussion needed to follow a different format and to compare our findings with other studies. We have developed the discussion and edited ensuring more extensive cross referencing to relevant literature. We have retained the section in implications for policy and practice as this is significant to the systematic review work in this project.

See all details in the attached file

Many thanks for providing very clear points in the attached file for us to follow. We have edited accordingly. We have retained the 1992 definition of sport as it is established and remains the citation used in the sport sector. We have made this clear in the text.

Reviewer 2

Thanks for the opportunity to review this paper. It is well written and deals with an important topic. Ways to positively influence the well-being of young people is crucial in promoting optimal mental and physical health. I was very pleased to see gray literature included.

The introduction could be strengthened with a stronger rationale for the review.
p. 3 is 'worthwhileness' a word?

We agree with the need for a stronger rationale for this important topic. We have edited the end of the introduction to include this text and cross reference to relevant literature.

“Interventions that positively influence the wellbeing of young people have the potential to promote good physical and mental health. [31-33] This review provides evidence that may improve understanding of the effects of sport and dance on a range of SWB measures and contribute to informing policy development, programme delivery and measurement and evaluation of sport and dance interventions to enhance wellbeing”

Worthwhileness is a word; a noun referring to the quality of being worthwhile

Methods:
Suggest a justification is provided for the timespan of the review.
Otherwise very comprehensive and rigourous section,

Many thanks for noting this. We have justified the time span as one which would allow us to reflect current and longer-term work on sport, dance and wellbeing

Results:
Well presented, easy to follow

Many thanks.

Discussion:
This section needs the most work. As written it is more like a summary of the results. These need to be clearly related to the broader literature. What does this all mean? How can this knowledge be utilised? How does it relate to what we already know?

We agree entirely and note that this comment is also made by reviewer 1. The discussion certainly needed to follow a different format and to compare our findings with other studies. We have developed the discussion and edited ensuring more extensive cross referencing to relevant literature. We have retained the section in implications for policy and practice as this is significant to the systematic review work in this project.

I encourage the authors to make these changes and good luck with your future work.

Many thanks for all expert reviews and support for this paper. We have made the changes and uploaded a document that shows the edits. In this revised manuscript we include edited tables (3 and 4) but with no track changes. We have uploaded tables 3 and 4 showing track changes in a different document as part of our response.

VERSION 2 – REVIEW

**REVIEWER**	Patrizia Calella<br>Parthenope University Naples
**REVIEW RETURNED**	09-Apr-2018

**GENERAL COMMENTS**	thanks to the authors for the responses. The new version of the manuscript is more clear and complete, also the tables now are easy to read and well defined. In my opinion there is no need for further revisions

**REVIEWER**	Brenda Happell<br>University of Canberra, Australia
**REVIEW RETURNED**	22-Apr-2018

**GENERAL COMMENTS**	Thank you for making the suggested changes. The paper is now greatly improved and makes an important contribution to the literature.
